# Black phosphorus-Au-thiosugar nanosheets mediated photothermal induced anti-tumor effect enhancement by promoting infiltration of NK cells in hepatocellular carcinoma

**DOI:** 10.1186/s12951-022-01286-z

**Published:** 2022-02-21

**Authors:** Changchang Jia, Fan Zhang, Jiamei Lin, Liwen Feng, Tiantian Wang, Yuan Feng, Feng Yuan, Yang Mai, Xiaowei Zeng, Qi Zhang

**Affiliations:** 1 Cell-Gene Therapy Translational Medicine Research Center, The Third Affiliated Hospital of Sun Yat-Sen University, Sun Yat-Sen University, Guangzhou, 510630 China; 2 School of Pharmaceutical Sciences (Shenzhen), Shenzhen Campus of Sun Yat-Sen University, Sun Yat-Sen University, No. 66, Gongchang Road, Guangming District, Shenzhen, 518107 Guangdong China; 3 School of Biomedical Engineering, Shenzhen Campus of Sun Yat-Sen University, Sun Yat-Sen University, No. 66, Gongchang Road, Guangming District, Shenzhen, 518107 Guangdong China; 4 Boji Medical Biotechnological Co. Ltd., Boji Pharmaceutical Research Center, Boji Medical Building, No. 62 Nanxiang First Road, Science City, Huangpu District, Guangzhou, 510000 China; 5 Department of Hepatobiliary Surgery, The Third Affiliated Hospital of Sun Yat-Sen University, Sun Yat-Sen University, Guangzhou, 510630 China; 6 Department of Medical Oncology, The Third Affiliated Hospital of Sun Yat-Sen University, Sun Yat-Sen University, Guangzhou, 510630 China

**Keywords:** Black phosphorus, Photothermal therapy, Cancer immunotherapy, Nanomedicine, Hepatocellular carcinoma

## Abstract

**Background:**

Hepatocellular carcinoma (HCC) is a heterogeneous cancer required combination therapy, such as photothermal therapy and chemotherapy. In recent years, cancer immunotherapies are rapidly evolving and are some of the most promising avenues to approach malignancies. Thus, the combination of the traditional therapies and immunotherapy in one platform may improve the efficacy for HCC treatment.

**Results:**

In this work, we have prepared a black phosphorus (BP)-Au-thiosugar nanosheets (BATNS), in which Au-thiosugar coating and functionalization improved the stability of both black phosphorus nanosheets (BPNS) and gold ions in different simulated physiological environments. The compression of the BATNS band gap can convert more photon energy to heat generation compared with BPNS, resulting in higher photothermal conversion efficiency. The in vitro and in vivo results also revealed a stronger reduction on the hepatocellular carcinoma of mice and prolonged survival of disease models compared with BPNS. More importantly, BATNS showed an additional immune effect by increasing local NK cell infiltration but not T cell on the liver cancer treatment, and this immune effect was caused by the thermal effect of BATNS photothermal treatment.

**Conclusions:**

The novel BATNS could improve the stability of BPNS and simultaneously combine the cancer thermotherapy and immunotherapy leaded by local NK cell infiltration, resulting in a better therapeutic efficacy on hepatocellular carcinoma. This work also provided a new path to design BP-based materials for biomedical applications.

**Graphical Abstract:**

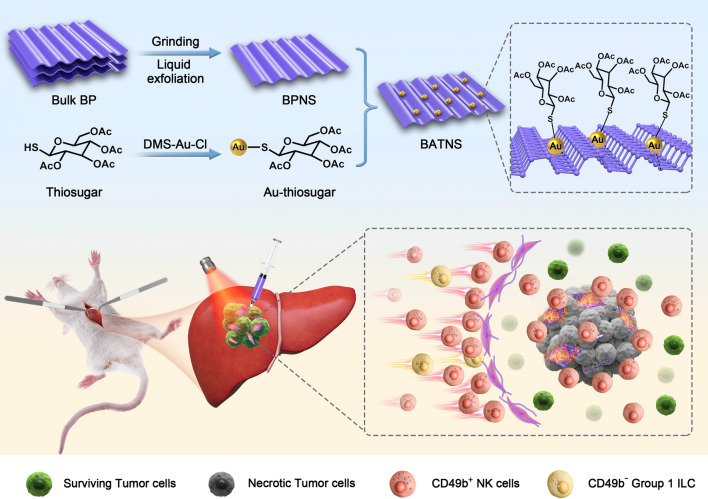

**Supplementary Information:**

The online version contains supplementary material available at 10.1186/s12951-022-01286-z.

## Background

There has been a long and traditional history to treat various pathologies with gold elements and compounds [1–3]. In the early twentieth century, sodium aurothiomalate and aurothioglucose, known as parenteral gold compounds, were first reported to be effective therapeutic compounds for rheumatoid arthritis (RA) [4–6]. Since that, gold compounds subsequently became the main treatment for RA, till a novel oral gold-containing drug was supported with a safety profile and was following approved by the U.S. Food and Drug Administration (FDA) in 1985 [7–9]. Auranofin (Additional file 1: Fig. S1) is a synthetic gold agent in which the central atom of gold is covalent bound with a phosphine ligand and a sulfur ligand, the former is responsible for its lipophilic character while the latter provides its inhibition on enzymes. Recently, auranofin has been receiving increasing attention to be applied in cancer treatment owing to its induction on the level of reactive oxygen species (ROS) and the signaling pathways inhibition involved in cancer progression [10, 11]. Also, auranofin is a powerful inhibitor of mammalian thioredoxin reductases (TrxRs), which are selenium-containing flavoenzymes over expressed in various cancer cells (such as human hepatoma carcinoma cell) [12] and possibly due to tumor cell proliferation and growth [13, 14]. Subsequently, auranofin-induced inhibition of TrxRs reduces the Trx reduced form and induces the Trx oxidized form in cells, resulting in cell death [15]. Based on this, recent studies have suggested that hepatoma carcinoma could be treated with auranofin in sufficient concentrations [16–19]. However, satisfied anti-cancer therapeutic efficacy was hardly observed with chemotherapy-only strategies.

In recent years, immunotherapy approaches are rapidly evolving [20], which has been used as a supportive therapy for cancer and combination with other therapies, such as chemotherapy would significantly improve the clinical outcomes of cancer treatment [21]. Gold nanoparticles (Au NPs) and/or gold ions had been reportedly caused specific immune effects [11], and Au NPs has been suggested as a potentially effective therapeutic and/or functional carrier in immunotherapies due to its beneficial features including customizable size and shapes, surface functionality and biocompatibility [22–24]. Thus, gold elements (such as auranofin) might have a dual therapeutic efficacy on cancer treatment due to their chemotherapeutic and immunotherapeutic features. However, neither Au NPs nor gold compounds has their limitations on the immunological treatment for hepatocellular carcinoma. On one hand, a few of gold ion could be released from Au NPs in tumor environment resulting in limited immune cell raising and immunotherapeutic efficacy. On the other hand, gold in ion form demonstrated a low bioavailability and high metabolism, which leading to a low level of gold ion distribution in the body [25]. Thus, a controlled and biocompatible material is being required to carry the gold ion targeting to the tumor, thereby showing the effects of gold ion-controlled immunity and chemotherapy.

Black phosphorus nanosheets (BPNS), a metal-free layered semiconductor, has been successfully serve as effective photothermal therapy (PTT) agents for the treatment of cancer [26], which can also be utilized as a nano-carrier for anti-tumor drugs delivery owing to its huge surface area for drug loading. However, BPNS could easily react with oxygen and water, subsequently degrade in air-exposed water solution [27]. Thus, surface covalent or noncovalent modification with small molecules were reportedly capable of stabilizing BPNS [28, 29]. In a recent study, Ag^+^ was observed to stabilize BPNS in air and realize the delivery of metal ion in one strategy. Briefly, Ag^+^ was spontaneously adsorbed on the surface of BPNS via cation–π interactions, thereby passivating the lone-pair electrons of P atoms in BPNS [30].

According to the above information, we hypothesize that loading Au-thiosugar on the surface of BPNS through the cation–π interaction could not only maintain the original structure of BPNS but also improve the stability of materials, while BPNS could be a suitable delivery system for auranofin. Moreover, thiosugar can ameliorate the biocompatibility of BPNS without additional chemical modification on the surface of BPNS. Thus, combinational platform of BPNS and auranofin (BATNS) could overcome the shortcomings of BPNS and auranofin but retains their advantages, along with the combination therapy on cancer, which potentially improve the therapeutic efficacy. Moreover, an increasing number of literatures have reported that various stress proteins (such as heat shock protein, calreticulin and high mobility group protein B1) could be generated under PTT environment and improved the immunogenicity of tumor cells [31–34]. Therefore, the combination of PTT and BATNS application might show a better tumor ablation and a stronger induction on host immune response. Indeed, our findings showed BATNS revealed a stronger reduction on the liver tumors of mice and prolonged their survival than BPNS, owing to its higher photothermal conversion efficiency. Furthermore, compared with BPNS, BATNS showed an additional immune effect by increasing local NK cell infiltration on the liver cancer treatment, providing a novel strategy for combining the cancer thermotherapy and immunotherapy and a new path to design BP-based materials for biomedical applications.

## Materials and methods

### Materials

Bulk black phosphorus (BP), 1-methyl-2-pyrrolidinone (NMP), (dimethylsulfide)gold(I) chloride, 1-Thio-*β*-d-glucose tetraacetate, dichloromethane and diethyl ether were purchased from Sigma-Aldrich.

### Preparation of BP nanosheets (BPNS)

Bulk black phosphorus (40 mg) was grinded to fine powder in a mortar and mixed with 40 mL of 1-methyl-2-pyrrolidinone (NMP). The mixture was degassed under argon atmosphere (5 min) and sonicated in ice bath for 27 h with a sonic tip (ultrasonication power: 50% amplitude (700 W), On/Off cycle: 5 s/5 s). The resulting solution was centrifuged for 10 min (5000 rpm) to remove the rest bulk BP. The supernatant was collected and centrifuged with 15,000 rpm for 10 min to give the desired precipitate. The final product in 20 mL NMP was degassed under argon atmosphere and stored at − 20 °C.

### Preparation of Au-thiosugar

(Dimethylsulfide)gold(I) chloride (1 equiv.) and 1-Thio-β-d-glucose tetraacetate (1.1 equiv.) were dissolved in well degassed dichloromethane (1 mL). The mixture was stirred at room temperature for 12 h without light. The resulting product was collected by evaporation and centrifugation (7500 rpm, 5 min). The product was washed with diethyl ether (4 × 10 mL) affording the pure white powder (65%). The pure product was dried under vacuum and stored at − 80 °C.

### Preparation of BP-Au-thiosugar nanosheets (BATNS)

BPNS (1 mg), Au-thiosugar (2.5 mg) and triethylamine (10 μL) were dissolved in the 1 mL of THF. The mixture was stirred for 24 h. The final product BATNS was collected by centrifugation (14,000 rpm) for 5 min and washed with THF (3 × 10 mL).

### Characterization of BP-Au-thiosugar nanosheets (BATNS)

The morphologies of BPNS, BATNS were characterized by transmission electron microscope (TEM, JEM-2010HR, JEOL, Japan) operating at 120 kV. Atomic force microscopy (AFM) was performed on Bruker Diension Icon microscope. The size and zeta potential of BPNS, BATNS were measured by Malvern Zetasizer Nano ZS90. The structures of BPNS, BATNS were determined by Raman scattering, X-ray photoelectron spectroscopy (XPS, Axis HSi, Kratos Ltd., UK), UV–vis–NIR spectrophotometry (Lambda 950, PerkinElmer, UK), X-ray diffraction (XRD), Inductively coupled plasma atomic emission spectrometer (ICP-AES) and Micro infrared spectrophotometry. The surface morphology and elemental distributions were analyzed by scanning electron microscope (SEM) and energy-dispersive X-ray spectroscopy (EDS).

### Au-loaded capacity on BATNS

The Au content on BATNS was quantified by ICP-AES (PerkinElmer Optima 8300, USA). The sample was dried under vacuum and digested with a mixture of nitric acid and hydrochloric acid (HNO_3_: HCl = 1:3 v/v). The digestion was further diluted to appropriate concentration for Au detection and the concentration was converted to Au mass percentage on BATNS.

### Stability capacity

The stability of BPNS, BATNS were studied by UV–vis–NIR spectrophotometry and transmission electron microscope. The UV–vis absorbance of the BPNS, BATNS dispersed in ultrapure water were monitored at 0, 1, 2, 3, 4, 8, 12, 24, 48, 72, 96, 120 h. To evaluate the influence of Au-thiosugar coating on BPNS stability, BPNS and BATNS were dispersed in pure water and exposed to air for 7 days and then their particle size were monitored at predetermined time intervals.

### Degradation of BATNS

BATNS were dispersed in a range of solutions (pH 7.4 aqueous solution, pH 6.8 aqueous solution, pH 5.3 aqueous solution and 10 mmol GSH aqueous solution for 24 h, pH 5.3 + 10 mmol GSH PBS solution for 72 h), and in pH 7.4 aqueous solution with laser to evaluate its stability. The morphology change of BATNS were observed by TEM.

### Photothermal property

To investigate the property of BATNS as photothermal agent, a range of concentrations of BATNS (25, 50, 100, 200 µg/mL) were irradiated by NIR laser at 1.0 W/cm^2^ for 10 min. 50 μg/mL BATNS was illuminated with different laser (0.5, 1.0, 1.5, and 2.0 W/cm^2^, 808 nm) for 10 min. Temperature change was recorded by a Ti450 Thermal Imager (Fluke, USA).

### Photothermal conversion efficiency

BPNS, BATNS and pure water were exposed to 808 nm laser (1 W/cm^2^) respectively until the temperature reached equilibrium, and then the laser was turned off. The heating and cooling curves of each sample were recorded using an infrared imager.

According to the Roper algorithm, the formula for calculating the photothermal conversion efficiency are as follows.

When conducting in vitro heating experiments, there are the following rules:

1 $$ \sum_{i} m_{i} C_{p,i} \frac{dT}{{dt}} = Q_{NPs} + Q_{s} - Q_{loss} . $$

In formula (1), T is the temperature of the system (℃), *Q*_*NPs*_ is the energy absorbed by nanoparticles (J), *Q*_*s*_ is the energy absorbed by the solvent (J), and *Q*_*loss*_ is the energy lost by the system (J).

According to the laws of thermodynamics, nanoparticles and solvents absorb energy during the heating process, and the system loses energy as follows:

2 $$ Q_{NPs} = I\left( {1 - 10^{{ - A_{\lambda } }} } \right)\eta , $$

3 $$ Q_{loss} = hA\Delta T, $$

4 $$ Q_{s} = Q_{loss} = hA\Delta T_{{max,{\text{H}}_{2} {\text{O}}}} , $$

5 $$ Q_{NPs} + Q_{s} = Q_{loss} = hA\Delta T_{max,mix} . $$

And according to Roper’s formula, the conversion efficiency can be obtained as:

6 $$ \eta = \frac{{hA\Delta T_{max,mix} - hA\Delta T_{{max,{\text{H}}_{2} {\text{O}}}} }}{{I\left( {1 - 10^{{ - A_{\lambda } }} } \right)}} = \frac{{hA\left( {\Delta T_{max,mix} - \Delta T_{{max,{\text{H}}_{2} {\text{O}}}} } \right)}}{{I\left( {1 - 10^{{ - A_{\lambda } }} } \right)}}, $$

wherein *η* is the photothermal conversion efficiency, h is the heat transfer coefficient of the container (W/(m^2^ ℃)), *A* is the surface area of the container (m^2^), *I* is the laser power (W), and *A*_*λ*_ is the absorbance of the nanoparticles at 808 nm.

Introduce θ, defined as the ratio of $\Delta T\;{\text{to}}\;\Delta T_{max}$:

7 $$ \theta = \frac{\Delta T}{{\Delta T_{max} }}. $$

We can obtain the following formula:

8 $$ \frac{d\theta }{{dt}} = \frac{{Q_{NPs} + Q_{s} - Q_{loss} }}{{\sum_{i} m_{i} C_{p,i} \Delta T_{max} }} = \frac{hA}{{\sum_{i} m_{i} C_{p,i} }}\left( {\frac{{Q_{NPs} + Q_{s} }}{{hAT_{max} }} - \theta } \right). $$

During the cooling process, the energy absorbed by the system is 0, so:

9 $$ Q_{NPs} + Q_{s} = 0, $$

10 $$ dt = - \frac{{\sum_{i} m_{i} C_{p,i} }}{hA}\frac{d\theta }{\theta }, $$

11 $$ t = - \frac{{\sum_{i} m_{i} C_{p,i} }}{hA}In\theta . $$

Since the little mass of solute, it was ignored in the calculation, i.e., $\sum_{i} m_{i} C_{p,i} = m_{{{\text{H}}_{2} {\text{O}}}} C_{{{\text{H}}_{2} {\text{O}}}} .$

hA can be determined by the linear relationship between the time t and the negative logarithm of the cooling cycle − Inθ. Fit θ according to formula (11), and get the slope of the curve as the value of ℎ*A*.

*A*^−*λ*^_808nm_ can be obtained according to the ultraviolet–visible spectrum analysis of the nanoparticles’ dispersion, and the laser power was 1 W.

According to formula (6), the photothermal conversion efficiency of the nanoparticles can be obtained.

### UV–Vis diffuse reflectance spectra for BPNS and BATNS

UV–vis DRS were performed on a Shimadzu UV-3600 Spectrometer using BaSO_4_ as background. Based on Kubleka-Munk theory, band gap of BPNS and BATNS were calculated. The reflectance data from Kubelka–Munk equation F(R) = α = (1 − R)^2^/2R, in which R is the percentage of reflected light has been guided the calculations of the optical absorption coefficient (α).The incident photon energy (*hν*) and the optical band gap energy (*Eg*) are related to the transformed Kubelka–Munk function, [F(R)*hν*]^2^ = A(*hν* − *Eg*), where *Eg* is the band gap energy, A is the constant depending on transition probability, and intercept of [F(R)*hν*]^2^ vs. *hν* is *Eg*.

### Valence band-XPS(VB-XPS) spectra of BPNS and BATNS

X-ray photoelectron spectroscopy was performed on an X-ray photoelectron spectroscopy (Axis HSi, Kratos Ltd., UK) with Al Kα radiation (1486.6 eV photons, 150 W) as the X-ray source for excitation. Detailed band structures and absolute band gaps were calculated from data of VB-XPS and UV–vis DRS. Precise position of the valance bands were revealed by VB-XPS.

### Cell culture

Mouse liver cancer cells Hep 1–6 were purchased from the American Type Culture Collection (ATCC). Hep 1–6 cells were cultured in DMEM medium (high glucose) with fetal bovine serum (10%, v/v), penicillin (1%, v/v) in a humidified incubator at 37 °C with 5% CO_2_ atmosphere.

### Cellular uptake

Hep 1–6 cells were seeded on laser confocal dish at a density of 20,000 cells/well and cultured for 24 h. The cells were incubated with fresh media containing DOX@BATNS at the concentration of 25 μg/mL for different times (0, 2, 4, 8, 12 h). Subsequently, the cells were washed with cold PBS for three times, fixed by 4% paraformaldehyde for 15 min at 4 °C and stained with DAPI (4 μg/mL, 10 min) at room temperature. Finally, the cells were soaked in 0.5 mL of PBS and imaged under confocal laser scanning microscopy (CLSM, LSM880, Zeiss, GER) to visualize the uptake of DOX@BATNS.

For flow cytometric analysis, Hep 1–6 cells were seeded into 6-well culture plates at 1 × 105 cells/well and cultured for 24 h. The cells were treated with fresh media containing 50 μg/mL DOX@BATNS for a range of times (0, 2, 4, 8, 12 h). After removing suspension, the cells were washed with PBS (3 × 1 mL), digested by trypsin. Finally, the cells were collected by centrifugation and analyzed by flow cytometer (CytoFLEX, Beckman, USA).

In vitro toxicity studies of BPNS and BATNS.

Hep 1–6 cells were seeded into 96-well culture plates at the density of 5000 cells/well and cultured for 24 h. Then the cells were incubated with BPNS and BATNS at different concentrations (0, 25, 50, 100 and 200 µg/mL) for another 24 h. Finally, the cell viabilities were measured by the CCK8 assay.

To investigate photothermal cytotoxicity of BPNS and BATNS, the cells were cultured in 96-well culture plates (5000 cells/well) for 24 h and incubated with PBS, BPNS and BATNS at different concentration (0, 25, 50, 100, and 200 µg/mL) for 8 h. Then the cells were irradiated with 808 nm laser (1 W/cm^2^) for 10 min. After irradiation, the cells were further cultured for 24 h, and the viability of the cells was evaluated using the CCK8 assay. The absorption was recorded at 450 nm by a microplate reader.

### Cell apoptosis

Apoptosis assays were conducted according to the manufacturer’s protocol for the annexin V-FITC apoptosis detection kit (KeyGEN BioTECH). Hep 1–6 cells were seeded into 6-well culture plates at 1 × 105 cells/well and cultured for 24 h. The cells were treated with fresh media containing nanoparticles at the concentration of 100 μg/mL. After incubating for 8 h, the PBS + NIR, BPNS + NIR, BATNS + NIR groups were exposed to an 808 nm laser irradiation at a power intensity of 1 W/cm^2^ for 5 min. After irradiation, the cells were further cultured for 6 h and subsequently evaluated using an annexin V-FITC apoptosis detection kit (KeyGEN BioTECH). The results were analyzed and obtained by a flow cytometer.

### Evaluation of antitumor effects by magnetic resonance imaging (MRI)

Male mice (4–6 weeks old, C57BL/6J) of all groups were recorded the MRI images of tumor regions at 21 days of treatments by a 9.4 T MRI scanner. The necrotic degree of tumor tissues was evaluated by the indexes of standard ADC, fast ADC and slow ADC.

### In vivo orthotopic tumor xenograft study

Male mice (4–6 weeks old, C57BL/J) were purchased from the Laboratory animal research institute (Keygen, China). The mouses were anesthetized with isoflurane and 1 × 106 Hep1-6 cells in 30 µL matrigel (BD, USA) were inoculated into the left lateral liver lobe. Orthotopic tumor of mouses were allowed to grow for about 1 week, and the tumor volumes were evaluated by MRI, subsequently the local tumor sites were injected nanosheets (BPNS and BATNS, 30 mg/kg) with or without irradiated with 808 nm NIR laser at a power density of 1.0 W/cm^2^ for 10 min. During the irradiation, an infrared thermal image camera was used to monitor the temperature changes. After 21 days the tumor volumes were again evaluated by MRI, and then the tumor tissue collected for flow cytometry and histologic study. All experiments involving animals were performed in accordance with ARRIVE (Animal Research: Reporting In Vivo Experiments) guidelines of the UK and were approved by The Institute Research Medical Ethics Committee of Sun Yat-Sen University.

### Flow cytometry

Mice were killed and orthotopic tumor xenograft of liver were harvested and prepared for flow cytometry as previously described [35]. For liver and tumor samples, lymphocytes were isolated by Ficoll density gradient centrifugation. Single cell suspensions from tumors and liver were obtained by tissue digestion as described [36] previously. Reagents or antibodies targeting the following epitopes were purchased from BioLegend: CD3 (145-2C11), CD19 (6D5), CD49b (DX5), NK1.1 (PK136), NKp46 (29A1.4), TCRβ (H57-597), CD4 (M5E2), CD8 (SK1), CD45 (104). Antibodies targeting CD49a (Ha31/8) were purchased from BD Biosciences. Cell number was calculated by using BD Liquid Counting Beads (BD Biosciences). A lineage (Lin) cocktail consisting of antibodies to CD3, CD19 and TCRβ (all identified above) was used for lineage-cell exclusion where indicated. Cells were washed once by PBS and then stained with fluorochrome-conjugated antibodies against surface markers for 30 min at 4 °C. Data acquisition was performed using LSRFortessa Flow Cytometer (BD Biosciences). Flow cytometry was performed using Flowjo (Treestar) software.

### Immunohistochemistry and HE staining

The tissue sections were dewaxed and debenzylated according to previous study [37]. and then incubated with rabbit anti-Ki67 (Abcam, USA), rabbit anti-HIF-1α (Abcam, USA), rabbit anti-NCR1 (Abcam, USA) and rabbit anti-CD3 (CST, USA) antibodies overnight at 4 °C. After washing, the bound antibodies were detected using horseradish peroxidase-conjugated secondary antibody (DAKO, Denmark) and diaminobenzidine (DAKO, Denmark), followed by counterstaining with hematoxylin (Keygen Biotech, Nanjing, China). The images were viewed under a fluorescence microscope (LEICA DMI 4000B, Solms, Germany) and were analyzed by Leica application suite software (version 4.0).

### Statistical analysis

The data are presented as the mean ± SD, and statistical comparisons of mean values were analyzed by one-way ANOVA using SPSS software (version 16.0). All the experiments were carried out at least in triplicate. Statistical significance was set at a P value < 0.05. Differences of P < 0.05 (*), P < 0.01 (**) was indicated.

## Results and discussion

### Synthesis and characterization of BATNS

**Scheme 1:**
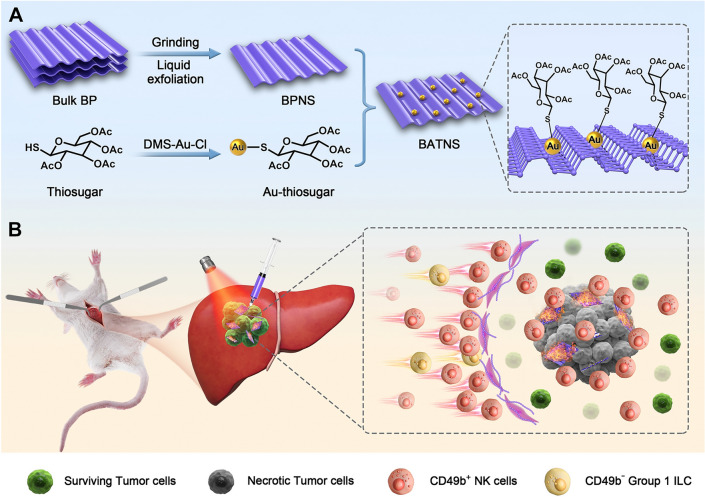
The schematic illustration of BP-Au-thiosugar nanosheets (BATNS) as photothermal-induced tumor killing agent for HCC. **A** The synthesis of BATNS. **B** The schematic illustration of BP-Au-thiosugar nanosheets (BATNS) as photothermal-induced tumor killing agent induce the increase of local NK cell infiltration caused by the thermal effect of BATNS photothermal treatment for HCC

**Fig. 1:**
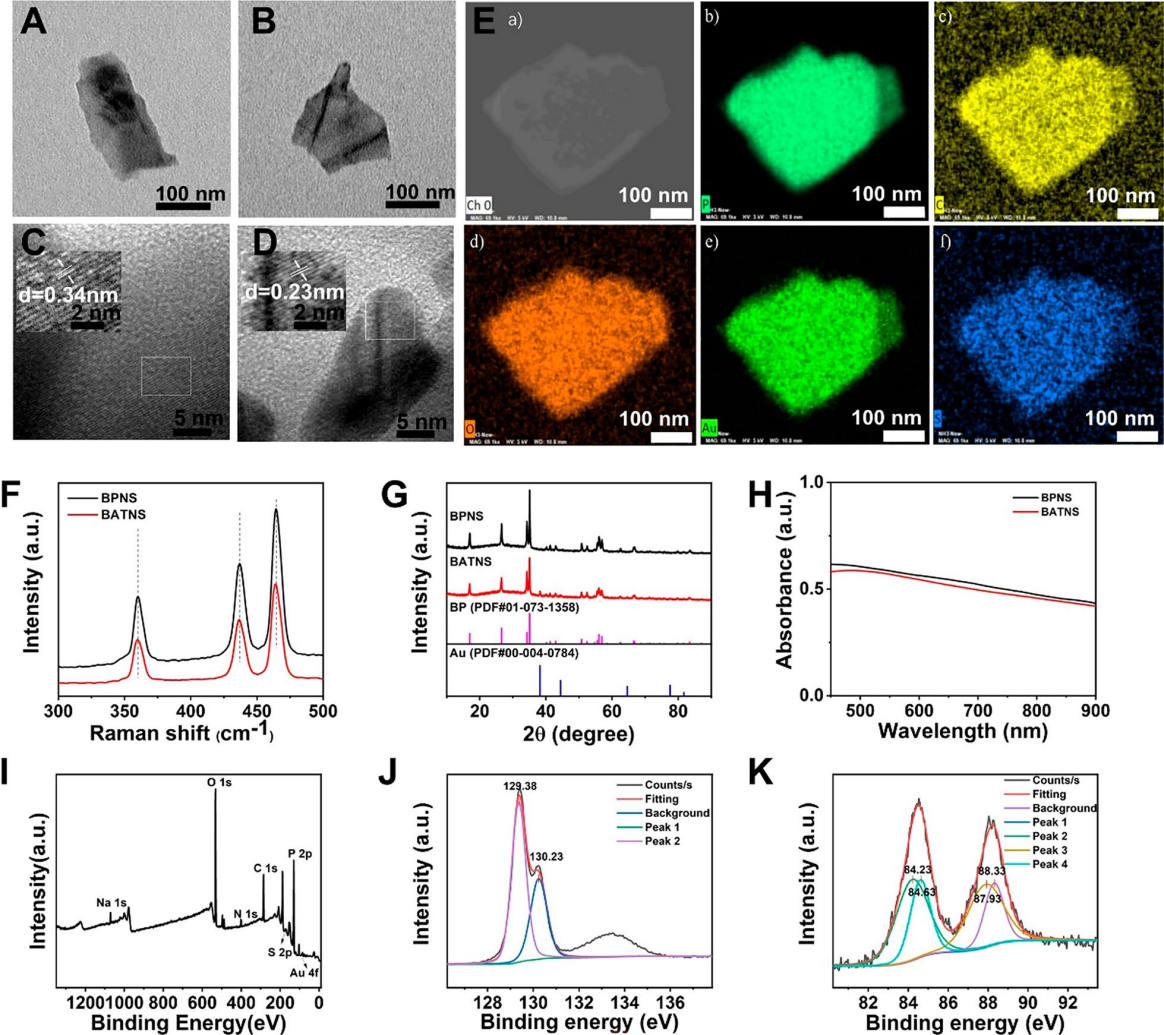
Characterizations of BPNS and BATNS. **A** TEM image of BPNS. **B** TEM image of BATNS. **C** HR-TEM image of BPNS. **D** HR-TEM image of BATNS. **E** SEM–EDS mapping of BATNS. **a** SEM image of BATNS, **b**–**f** EDS element mapping images of BATNS for P, C, O, Au and S. **F** Raman spectra of BPNS and BATNS. **G** XRD analysis of BPNS and BATNS. **H** UV–vis absorption spectra of BPNS and BATNS. **I** XPS spectra of BATNS. **J** XPS spectra of P 2p for BATNS. **K** XPS spectra of Au 4f for BATNS

The synthesis approach of BATNS is illustrated in Scheme 1, in which the preparation of BPNS was based on grinding and liquid exfoliating method as described in the literature, followed by Au-thiosugar surface modification with Au-P coordination bond [38–42]. The loading rate of Au on the BPNS (1.65%) was determined via ICP-AES. The transmission electron microscopy (TEM) images of prepared BPNS and BATNS are shown in Fig. 1A, B. Both of BPNS and BATNS present a complete two-dimensional sheet structure with the size of 187.7 ± 8.2 nm and 158.1 ± 6.3 nm, respectively (Additional file 1: Fig. S2). The Zeta potential of BPNS was − 30.55 mV, and BATNS was − 19.9 mV, indicating the successful loading of gold cation on the surface of BPNS (Additional file 1: Fig. S3). The AFM images revealed that the thickness of BPNS and BATNS were both about 1.5 nm (Additional file 1: Fig. S4). The 0.34 nm interplanar spacing for prepared BPNS can be observed in the corresponding high-resolution TEM (HR-TEM) image and matched with the (0 2 1) plane of P. Compared with BPNS, the interplanar spacing of BATNS was reduced to 0.23 nm, which matched with the (0 4 1) plane of P. This result shows that the loading of Au-thiosugar changed the exposed crystal plane of BPNS (Fig. 1C, D). Following the SEM–EDS images, the surface of BATNS could be observed to remains smooth, and the C, S, Au, P, O elements were uniformly distributed on BPNS, which confirmed the successful attachment of Au-thiosugar on the surface of BPNS (Fig. 1E, Additional file 1: Fig. S5). Raman scattering was further employed to characterize the BPNS and BATNS. Three Raman peaks of BPNS at 357.2, 432.4 and 460.5 cm^−1^, representing one out-of-plane mode ($A_{g}^{ 1}$) and two in-plane modes ($B_{g}^{ 2}$ and $A_{g}^{2}$), can be obviously observed in Fig. 1F. The Raman spectra of BATNS showed nearly the three peaks located at the same positions with those of BPNS, indicating that the loading of Au-thiosugar could maintains the complete structure of BPNS. The crystal structure of prepared BPNS and BATNS were determined by X-ray diffraction (XRD). The XRD pattern showed in Fig. 1G, the diffraction peaks of synthetized BATNS were matched with BPNS, the black phosphorus standard pattern and Au standard pattern, indicating that Au-thiosugar has been loaded successfully on the surface of BPNS without the change of the crystal structure of BPNS. As shown in Fig. 1H, the representative absorption of BPNS and BATNS can be observed from 450 to 900 nm. Both BPNS and BATNS have a strong light absorption capacity in the window of visible light and NIR-I. For the XPS test (Fig. 1I–K), after Au-thiosugar coating and functionalization, the new peaks of C, O, S and Au were presented in XPS spectrum of BATNS. The two strong peaks at 129.38 eV and 130.23 eV corresponded to P 2p_3/2_ and P 2p_1/2_ doublets from the BPNS, respectively. The peaks of 84.2, 84.6 eV(4f_7/2_) 88.3, 87.87 eV(4f_5/2_) evidenced the presence of two chemical environments for Au atoms (Au, Au^+^), indicating the successful loading of the Au-thiosugar.

### Stability and photothermal performance of BATNS

**Fig. 2:**
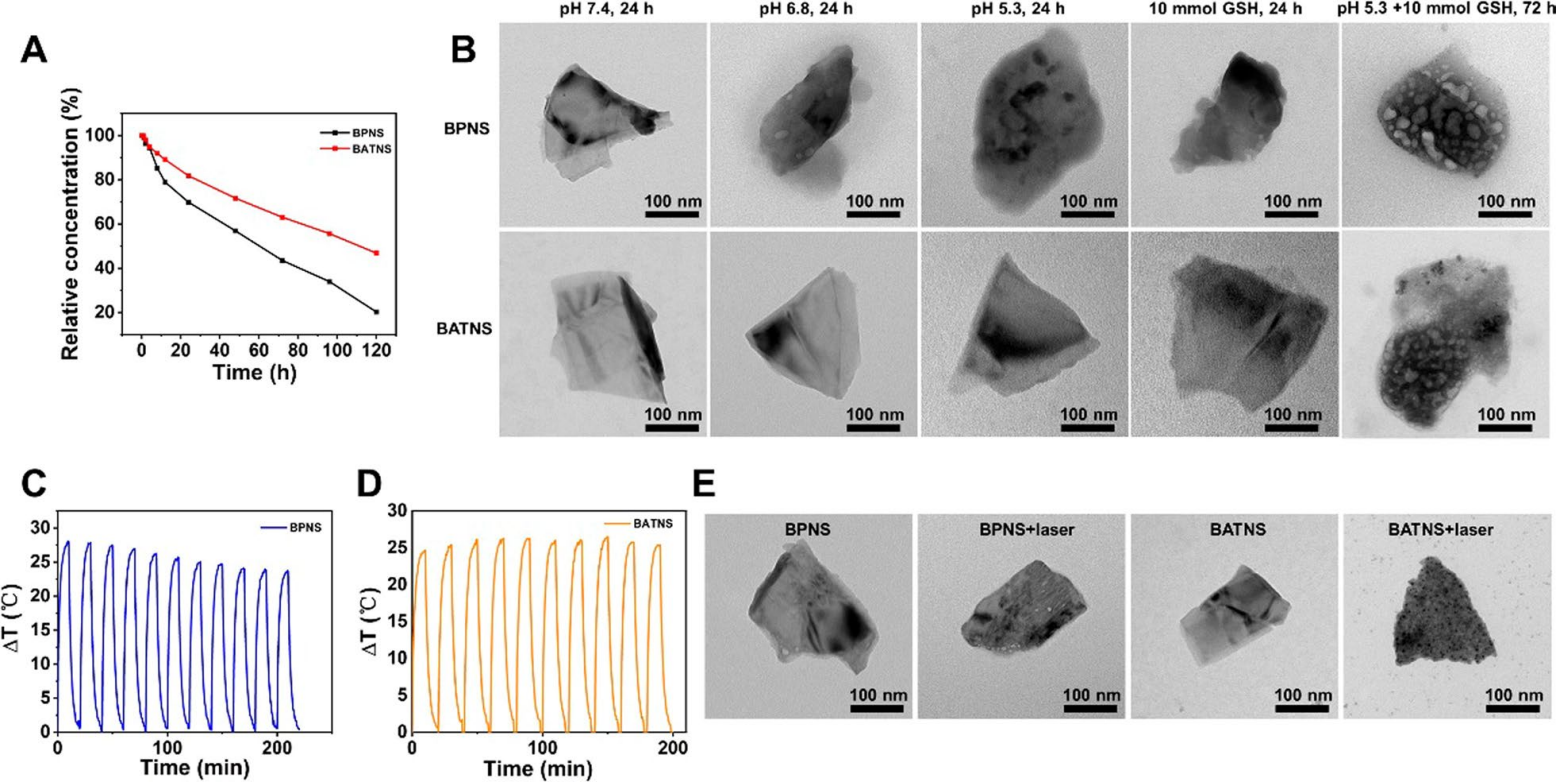
Stability of BATNS. **A** Degradation rate of BPNS and BATNS in water and air for 120 h. **B** TEM images of BPNS and BATNS dispersed in pH 7.4 PBS, pH 6.8 PBS, pH 5.3 PBS, 10 mmol GSH for 24 h and pH 5.3 + 10 mmol GSH for 72 h. **C**, **D** Temperature variation of the suspension of BPNS and BATNS in water over ten laser on/off cycles under 808 nm NIR irradiation (1.0 W/cm^2^). **E** TEM images of BPNS and BATNS before and after 808 nm NIR irradiation (1.0 W/cm^2^)

The application in vivo of nanomedicine with a size between 20 and 200 nm has the advantages of the enhanced permeability and retention effect, which can improve the accumulation in tumor and prevent the rapid renal filtration. Nevertheless, the unsatisfactory biodegradability and the long-term retention of inorganic nanomaterial in vivo have always been the main problems of its application. Therefore, the research of controllable biodegradability which not only ensures efficient tumor targeting and accumulation, but also avoids long-term retention, is the basic clinical demands for nanomedicine application. At first, the degradability of BPNS and BATNS in DI water were analyzed to evaluate the influence of Au-thiosugar coating on the surface of BPNS. The absorption spectra in Additional file 1: Fig. S6 shows that the absorbance intensity of BPNS almost decreased to zero, which means the BPNS was decomposed in DI water for 120 h. In the case of BATNS, the decrease of the absorbance intensity is less than that of BPNS. Comparing these data, we can observe less than 50% of BATNS was degraded, while BPNS was decomposed more than 80%. This phenomenon shows that loading of Au-thiosugar greatly enhanced the stability of BPNS (Fig. 2A). The hydrodynamic size of BPNS and BATNS was further monitored for a week (Additional file 1: Fig. S7). It can be observed that the sizes of these nanosheets had no significant change. This study demonstrated that BPNS and BATNS could maintain good photothermal stability in water for a week. Then, the stability of BPNS and BATNS were investigated in different simulated physiological environments by TEM. After treatment of BPNS in pH 7.4, pH 6.8, pH 5.3 and 10 mmol GSH for 24 h, the structures of BPNS can be found to destroy. In contrast to the situation of BPNS, the morphologies of BATNS still maintained its sheet structures without obvious aggregation or degradation, indicating the existence of Au-thiosugar improve the stability of BP, which revealed BATNS can better realize the effective accumulation in tumor. Moreover, both BPNS and BATNS have the capacity of self-degradation in simulated tumor micro-environment (TME) after 72 h (Fig. 2B). Phosphorus atoms provide chemically active sites for oxygen molecules due to their lone pair of electrons, making them easily be oxidized and degraded in air or water to result the rapid disappearance of the structure and properties of BP. This unstable phenomenon is particularly obvious in BPNS, so photothermal stability is one of important factors in the application of BPNS as photothermal material candidate. Hence, we studied the photothermal stability of BPNS and BATNS in DI water under 808 nm laser irradiation. As shown in Fig. 2C, D, the temperature of BPNS decreased slowly under laser irradiation, while the temperature of BATNS gradually stabilized after slowly raising. The TEM images in Fig. 2E show the morphology of BATNS have the negligible change after laser irradiation, demonstrating its high photostability. It is noting that some 2–3 nm nanoparticles can be observed on the surface on the BATNS due to the formation of gold nanoparticles after laser irradiation.

**Fig. 3:**
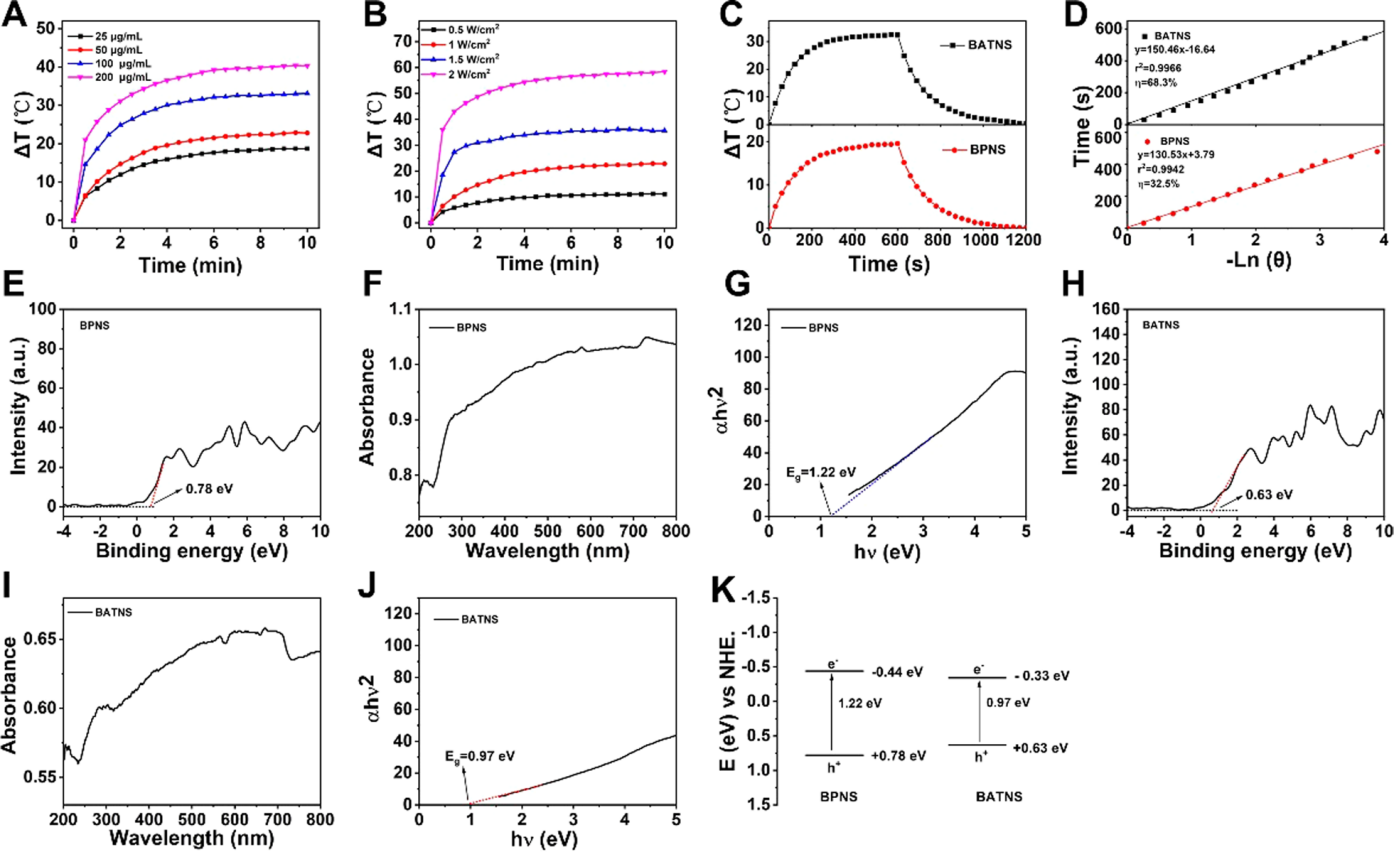
Photothermal mechanism of BATNS. **A** Temperature curves of BATNS solution at different concentration (25, 50, 100, 200 μg/mL) with 808 nm NIR laser irradiation (1.0 W/cm^2^, 10 min). **B** Temperature curves of BATNS solution (50 μg/mL, 10 min) under the 808 nm NIR laser irradiation of various power (0.5, 1.0, 1.5, 2.0 W/cm^2^). **C** The temperature increment curve and the cooling curve of BPNS and BATNS. **D** Line curve of time vs. − lnθ obtained from the cooling period of ). **E** The valance band of BPNS. **F** UV–vis reflectance spectrum of BPNS. **G** The band gap of BPNS estimated from UV–vis reflectance spectrum. **H** The valance band of BATNS. **I** UV–vis reflectance spectrum of BATNS. **J** The band gap of BATNS estimated from UV–vis reflectance spectrum. **K** Band structure of BPNS and BATNS

In next, the photothermal performance of BATNS was explored with various concentrations (25–200 µg/mL) and power density (0.5–2 W/cm^2^) at 808 nm. At 200 µg/mL of BATNS, the highest temperature change of 40.4 ℃ was achieved via 808 nm laser irradiation at 1.0 W/cm^2^ for 10 min (Fig. 3A, Additional file 1: Fig. S8). The photothermal conversion performance of BATNS at varied power density under 808 nm laser irradiation at the concentration of 50 µg/mL BATNS were conducted and exhibited in Fig. 3B and Additional file 1: Fig. S9. According to the published methods, the photothermal conversion efficiency (*η*) of prepared BPNS and BATNS reached to 32.5% and 68.3% under 808 nm laser irradiation (Fig. 3C, D). Following to the above result, we found that loading of Au-thiosugar on BPNS elevated the photothermal conversion efficiency of BATNS more than twice that of BPNS. To understand the mechanism of the enhanced photothermal conversion efficiency (*η*) of BATNS, its physical properties were investigated. The light absorption behavior of synthetized BPNS and BATNS were measured by UV/Vis diffuse reflectance spectroscopy (DRS) and ultraviolet photoelectron spectroscopy (VB–XPS), where the band gap (E_g_) and valence band (VB) of BPNS and BATNS were determined, respectively. As shown in Fig. 3E–J, BATNS exhibited a band gap of 0.97 eV, and the gap between frontier orbitals of BPNS was 1.22 eV, too wide to be excited at the same condition. Compared with conventional wide bandgap semiconductors, in which the light energy absorbed mostly reemits as photons after recombination of electron–hole pairs near the bandgap edge [43], in the case of BP materials, the above-bandgap electrons and holes can relax to the band edges and convert the extra energy into heat through a thermalization process. Therefore, under a same photon energy, the compression of the BATNS band gap can convert more photon energy to heat generation. This is the reason that BATNS has higher photothermal conversion efficiency than the BPNS (Fig. 3K).

### In vitro antitumor efficacy

**Fig. 4:**
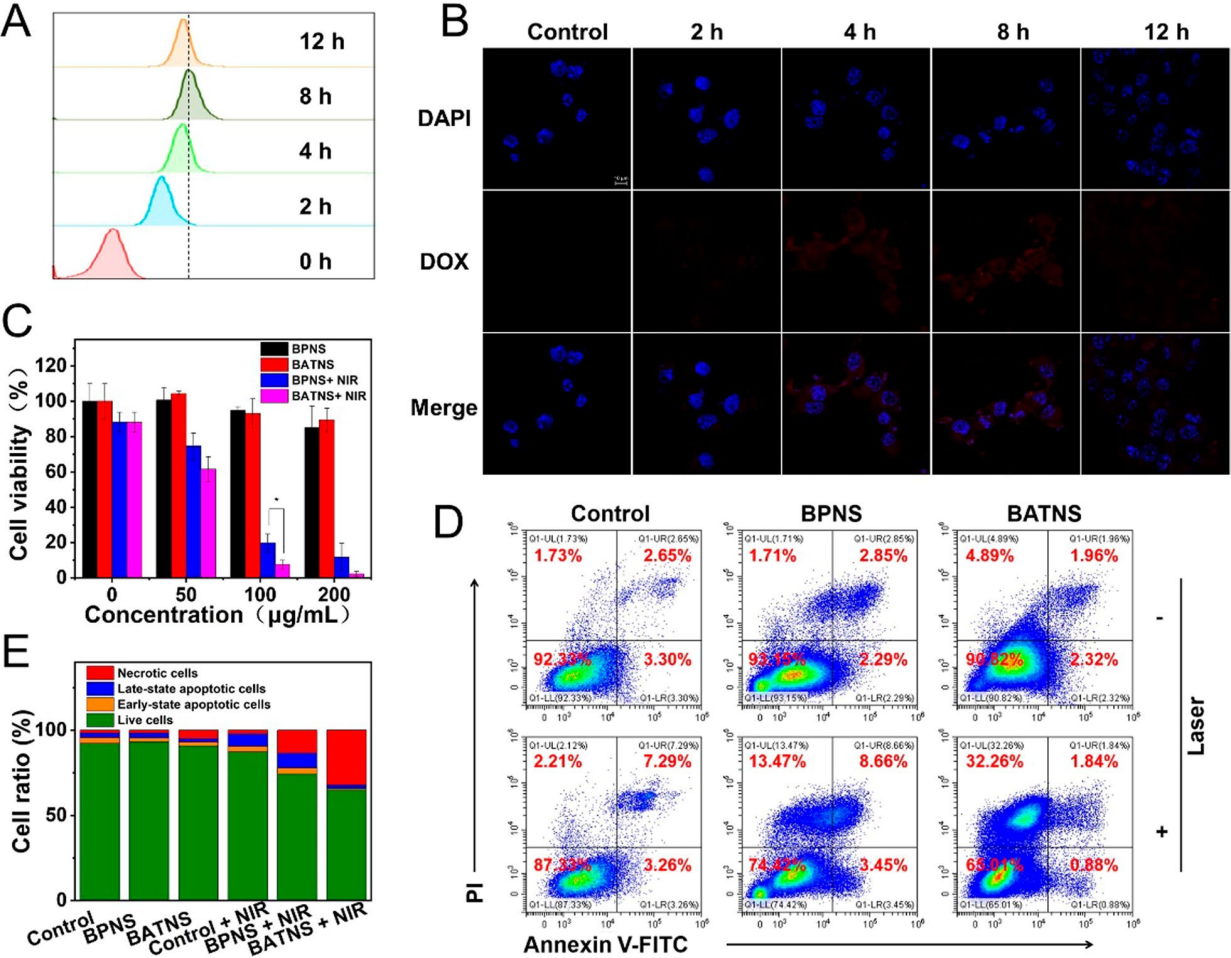
Anticancer efficacy of BATNS in vitro. **A** Cellular uptake analysis results of the same concentration of DOX labeled BATNS in Hep1-6 cells by flow cytometry analysis (50 μg/mL). **B** Fluorescence images of Hep1-6 cells treated with DOX labeled BATNS (25 μg/mL) and stained with DAPI (nucleus) for 0, 2, 4, 8 and 12 h, respectively. **C** The Hep1-6 cells viability of BPNS and BATNS at various concentrations with/without 808 nm NIR laser for 24 h (1 W/cm^2^, 10 min) (*p < 0.05). **D** Annexin V-FITC/PI double staining results under different treatment (100 μg/mL, 1 W/cm^2^, 5 min). **E** Histogram of apoptosis of Hep 1–6 cells detected by AnnexinV/PI staining matched with **D**

Biocompatibility is one of requisites for photothermal agents used in biomedicine. Firstly, we examined the capacity uptake of BATNS in the case of Hep 1–6 cells. DOX as a fluorescent marker was labeled on BATNS. Hepatoma cells Hep 1–6 were incubated with a medium containing 50 μg/mL BATNS@DOX for various times (0, 2, 4, 8, 12 h). The uptake of BATNS@DOX of Hep 1–6 were analyzed by flow cytometry. Figure 4A shows that the Hep 1–6 cells uptake of BATNS gradually increased within 8 h of administration, and then decreased at 12 h. This might be due to degradation and exocytosis of the BATNS in the acidic and GSH environment of tumor cells after being ingested, resulting in a decrease in fluorescence intensity at 12 h. Moreover, Laser confocal microscope results further certified that the prepared BATNS can be maximum taken up by Hep 1–6 cells into the cytoplasm at 8 h (Fig. 4B). The cytotoxicity and photothermal efficacy of BATNS were employed on the level of tumor cells Hep 1–6. Hep1-6 cells were incubated with different concentrations of BPNS and BATNS for 24 h. The results showed that both cell survival rate of BATNS and BPNS were above 85%, indicating that BATNS exhibited a great biocompatibility in normal physiological environment and a dose-dependent cytotoxicity for cancer cells. To investigate the photothermal cytotoxicity, Hep1-6 cells were incubated with different concentrations of BPNS and BATNS, each well was irradiated with 808 nm near-infrared light (1 W/cm^2^, 10 min), and then continued culturing for 24 h. BPNS and BATNS exhibited strong photothermal cytotoxicity on Hep1-6 cells at 100 μg/mL, the cell survival rate of the BPNS group was 19.78%, and the cell survival rate of the BATNS group was 7.61%, which exhibited statistically difference (p < 0.05). At a concentration of 200 μg/mL, the cell survival rate of the BPNS group was 11.76%, and the cell survival rate of the BATNS group was only 2.26%. These results obviously prove the satisfactory photothermal effect in vitro of the BATNS under NIR–I laser irradiation (Fig. 4C). To further study the photothermal effects of BATNS, we tried to investigate Hep 1–6 cells distribution proportion under laser irradiation. As shown in Fig. 4D, E, the dead cells proportion of BATNS combination with laser group remarkably enhanced to all other groups, proving that BATNS combined with laser irradiation induced most efficacious Hep 1–6 cells necrosis. Consequently, the BATNS with laser irradiation exhibits more phototherapeutic effects than BPNS, which could be explained by the great stability and high photothermal conversion efficiency of BATNS.

### Anticancer efficacy of BATNS-mediated PTT in vivo

**Fig. 5:**
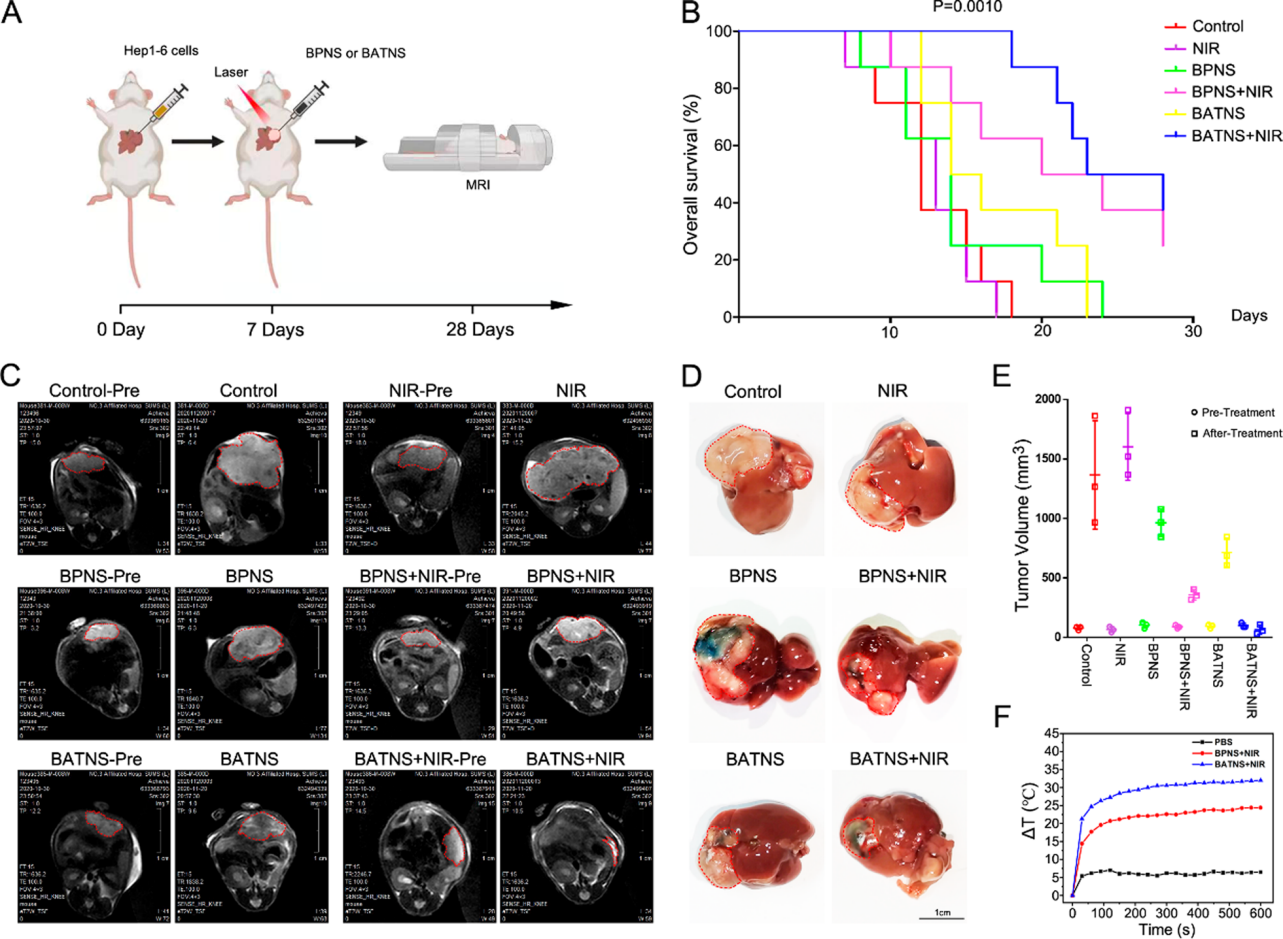
Anticancer efficacy of BATNS-mediated PTT in vivo. **A** Schematic illustration of BATNS combined with PPT to inhibit orthotopic liver tumor growth. **B** Survival curves of the mice in different groups (n = 8). Data was analyzed by the log-rank. **C** MRI imaging shows the contrast change between the maximum cross-section of the mouse tumor before treatment (Pre, the red dotted line shows the extent of tumor occupation) and after treatment (red dotted line). **D** Optical photo of tumors after 21 days of treatment (the red dotted line shows the extent of the tumor visible on the surface of the liver). **E** Statistical analysis of tumor volume changes calculated by MRI imaging (*p < 0.05, **p < 0.01). **F** Temperature change curves of the tumor region injected with PBS, BPNS, BATNS under 808 nm NIR laser (1.0 W/cm^2^, 10 min)

It is a very safe method to treat mouse tumors by local injection of nanosheets (BPNS and BATNS) and 808 nm infrared laser irradiation. Usually about 1 h after the operation the mice can return to normal walking. Before the operation, all the mice were tested by MRI (Fig. 5A). Through MRI, it was observed that the tumors of all experimental mice were confined to the liver and there was no significant difference in the size of the tumors in each group on MRI before surgery (Fig. 5E). After treatment, it is found that the tumors are not reduced by local laser treatment alone. Local injection of BPNS or BATNS (30 mg/kg) alone did not show a reduction in tumors compared with the pre-treatment, but the tumor growth was inhibited compared with the group of control and laser treatment. However, the tumor shrinkage after BPNS and BATNS injection followed by photothermal therapy was significant, and the effect of BATNS combined with photothermal therapy was the most significant (Fig. 5C, D). By analyzing the photothermal effect with a temperature detector, we found that the local laser treatment increased the temperature of local tumor 5 °C, while the highest temperature in the BP photothermal treatment group could rise 24 °C, and the BAT photothermal group could rise 32 °C (Fig. 5F and Additional file 1: Fig. S10), further analysis results of mouse survival also showed that the BPNS photothermal treatment group and BATNS photothermal treatment group significantly improved the mice survival (Fig. 5B).

### Immune responses after BATNS-mediated PTT in vivo

**Fig. 6:**
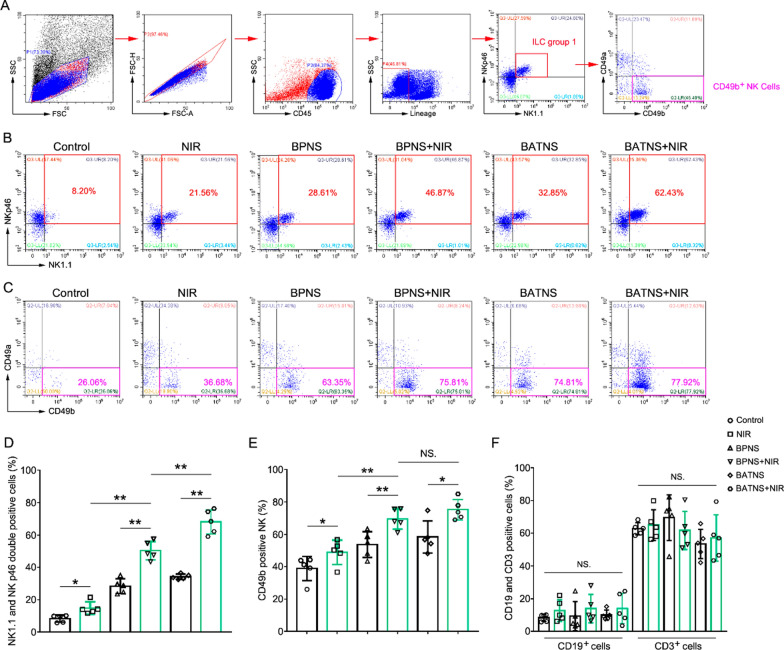
The local infiltrating NK cells of liver cancer significantly increased after BATNS photothermal treatment. **A** Flow cytometry analysis of the percentage of ILC group 1 subsets and the percentage of NK cells. **B** Representative quantitative flow cytometric analysis of NK1.1 and NKp46 double-positive cells (ILC group 1) in each experimental group. **C** Representative quantitative flow cytometric analysis of CD49b^+^ NK cells in ILC group 1 in each experimental group. **D** Statistical analysis of the percentage of the total ILC group 1 in each experimental group. **E** Statistical analysis of the percentage of CD49b^+^ NK cells in ILC group 1 in each experimental group. **F** Statistical analysis of the percentage of CD19^+^ B cells and CD3^+^ T cells in CD45^+^ lymphocytes in liver cancer tissues of mice in each experimental group (*p < 0.05, **p < 0.01)

**Fig. 7:**
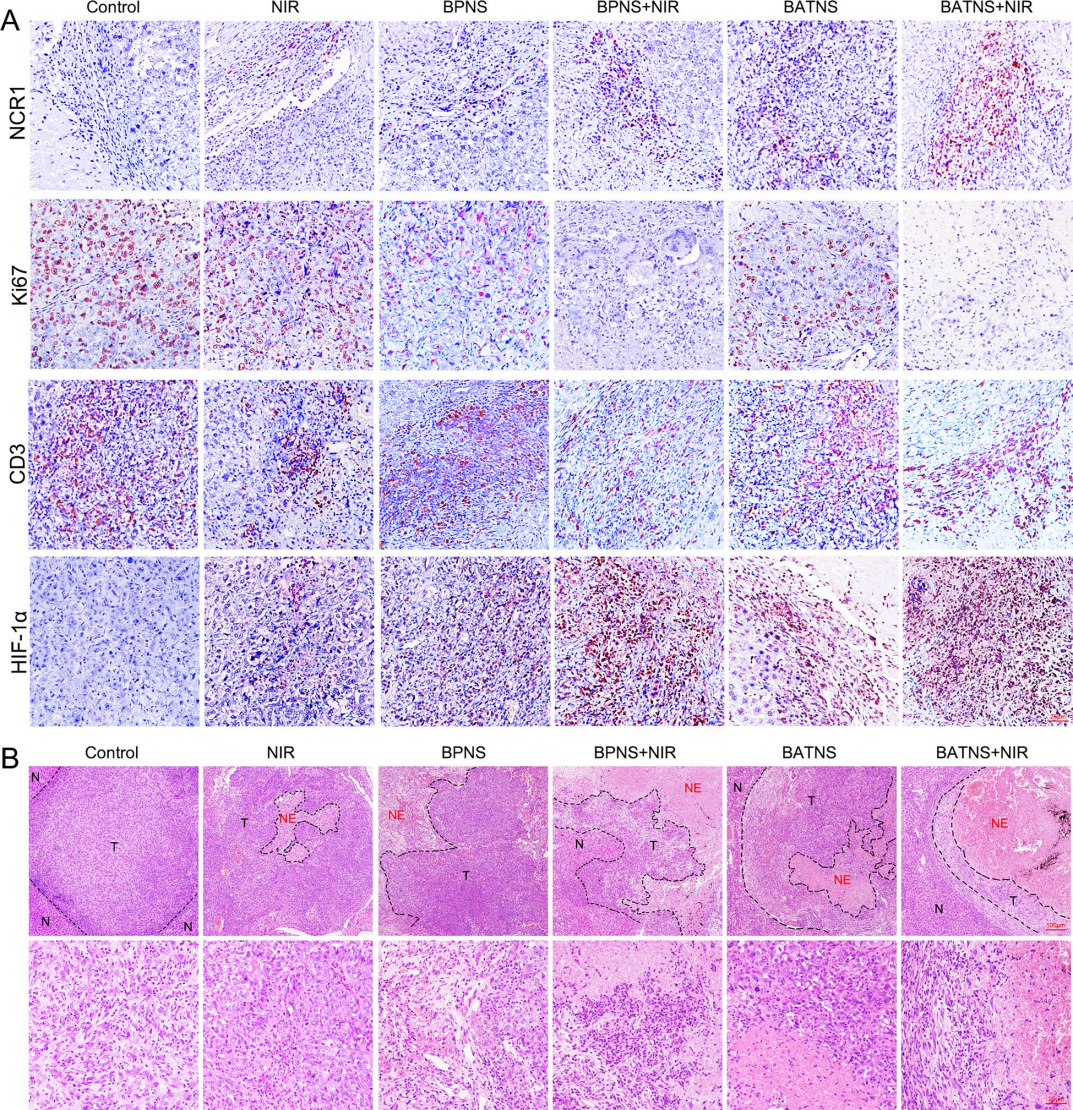
Analyze the changes of tumor histological indicators after BATNS photothermal treatment. **A** Immunohistochemical analysis of NK cells (NCR1), cell proliferation indicators (Ki67), T cells (CD3) and cellular hypoxia indicators (HIF-1α). **B** HE staining showed the local conditions of tumor tissue (T) adjacent to the cancer and necrotic tissue (NE) in the tumor in each experimental group. The tissue structure was observed under a ×100 microscope (up), and the bottom under a ×400 microscope

Researching the photothermal effect, in addition to killing tumors through direct thermal damage, it can also produce immune effects by activating tumor immunity to further kill tumors. At present, the immune effect induced by BATNS photothermal is not very clear, so we analyze the local immune cell components to explore the immune response induced by the photothermal effect of BATNS. Flow cytometry detected the local infiltration of immune cells in each treatment group, including T cells, B cells and NK Cells which were belongs to group 1 of innate lymphoid cells (ILC) (Fig. 6A and Additional file 1: Fig. S11A). The results show that T cells, B cells have no significant changes after BATNS photothermal treatment (Fig. 6F and Additional file 1: Fig. S11B), while NKp46^+^ and NK1.1^+^ double-positive ILC group 1 cells are significantly increased (Fig. 6B, D). Further analysis showed that NK cells infiltration increased significantly (Fig. 6C, E). Among ILC group 1 cells, CD49b^+^CD49a^−^ NK cells, which has a significant tumor-killing effect are the most significant increase, and such NK cells with high expression of granzyme, perforin and IFN-γ which were considered to the key to killing tumors [36, 44]. Further immunohistochemical analysis verified that NCR1^+^ NK cells were significantly increased in the BATNS photothermal group, while other immune cells such as CD3^+^ T cells did not change obviously. Simultaneously, we tested the cell proliferation marker Ki67 and indicators of hypoxia HIF-1α. The results showed that the number of Ki67^+^ positive cells in the BPNS group and especially in BATNS group was significantly reduced and expression of HIF-1α were also extremely the highest in the BATNS group (Fig. 7A). All the results demonstrated that BPNS photothermal treatment can increase the number of tumor NK cell infiltration and decrease the expression of tumor proliferation index Ki67 and increased tissue hypoxia, and BATNS photothermal treatment induced NK cell infiltration and the reduction of Ki67 and increasing of tissue hypoxia were the most significant, while the effects of BPNS or BATNS alone on NK cell infiltration and the reduction of Ki67 were not obvious. Furthermore, it can also be observed through HE that the tumor necrosis area in the BATNS photothermal group is significantly increased and the residual tumor cells are significantly reduced (Fig. 7B), suggesting that BATNS has the most significant tumor clearance effect after photothermal treatment.

Thermotherapy with radio-frequency ablation and microwave as the representations is becoming one of the first-line treatments for early-stage liver cancer, owing to its noninvasiveness, easy-handling, safety and high clinical value. However, ~ 80% patients were suffered from tumor recrudescence after thermotherapy [45]. Nanomaterials have recently been reported to active the immunity system and cause specific immune effects alongside the physical ablation by heat, suggesting their potential reduction on tumor recrudescence [46, 47]. Based on this, comprehensive investigations on the specific immune responses of nanomaterials on the liver cancer should be conducted. For the first time, we studied the thermotherapeutic efficacy of BPNS and its related nanomaterials (BATNS) on the in-situ liver tumor. Our findings showed both BPNS and BATNS demonstrated a therapeutic effect for the liver cancer treatment, while a better treatment was observed with BATNS compared with BPNS in terms of the tumor size and survive period. Moreover, a full profile of immune cells in tumors was obtained before and after the thermotherapy and a statistically significant change was observed in the quantification of NK cells. NK cells are key components of the innate immune system providing potent anti-tumor immunity and immune exclusion [36, 44], T cells however are always receiving more attentions in immunotherapy for liver cancer [48, 49]. Interestingly, we demonstrated herein the thermotherapy of BATNS increased NK cells instead of T cells to conduct the immune effects on cancer treatment. In addition, low infiltration of NK cells and T cells in solid tumor was a big challenge in cancer treatment. The immunohistochemical results in this study found that NK cellular infiltration in local tumor was improved after the application of both BPNS and BATNS. Thus, our findings suggested BATNS induced the infiltration of NK cells by thermo-effects providing a new solution in liver cancer treatment.

## Conclusion

In summary, we have designed a BP-Au-thiosugar nanosheets (BATNS) and demonstrated its enhanced stability, photothermal conversion efficiency and immunotherapy mechanism, excellent antitumor effects. Briefly, Au-thiosugar coating and functionalization improved the stability of BPNS in different simulated physiological environments. Also, the compression of the BATNS band gap can convert more photon energy to heat generation compared with BPNS, resulting in higher photothermal conversion efficiency. The photothermal effect of BATNS was further confirmed in vivo, which revealed a stronger reduction on the liver tumors of mice and prolonged their survival than BPNS. Furthermore, BATNS showed an additional immune effect on the liver cancer treatment compared with BPNS. The mechanism revealed that photothermal-induced tumor killing is related to the increase of local NK cell infiltration but not T cell caused by the thermal effect of BATNS photothermal treatment. This work provided a novel strategy for stabilizing BPNS and simultaneously combining the cancer thermotherapy and immunotherapy, paving a new path to design BP-based materials for biomedical applications.

## Supplementary Information

**Additional file 1: Figure S1.** The chemical structure of auranofin. **Figure S2.** DLS size distribution of BPNS and BATNS. **Figure S3.** Zeta potential of BPNS and BATNS. **Figure S4.** AFM images of BPNS (A) and BATNS (B). AFM measured thickness of BPNS (C) and BATNS (D). **Figure S5.** EDS spectrum of BATNS. **Figure S6.** UV–vis absorption spectra of BPNS and BATNS in water and air for 120 h. **Figure S7.** Stability of BPNS and BATNS in water, respectively, by monitoring particle size for 7 days. **Figure S8.** Infrared thermal image of BATNS solution at different concentration (25, 50, 100, 200 μg/mL) with NIR laser irradiation (1.0 W/cm^2^, 10 min). **Figure S9.** Infrared thermal image of BATNS solution (50 μg/mL, 10 min) under the 808 nm NIR laser irradiation of various power (0.5, 1.0, 1.5, 2.0 W/cm^2^). **Figure S10.** Infrared thermal images of the tumor region injected with PBS, BPNS, BATNS under 808 nm NIR laser (1.0 W/cm^2^). **Figure S11.** (A) Flow cytometry analysis of the percentage of CD4^+^ and CD8^+^ cells in total CD3^+^ T cells. (B) Statistical analysis of the percentage of CD4^+^ and CD8^+^ cells in T cells in each experimental group.

## Data Availability

All data generated or analyzed during this study are included in this article.
