# Correction to: Black phosphorus‑Au‑thiosugar nanosheets mediated photothermal induced anti‑tumor effect enhancement by promoting infiltration of NK cells in hepatocellular carcinoma

**DOI:** 10.1186/s12951-022-01412-x

**Published:** 2022-04-26

**Authors:** Changchang Jia, Fan Zhang, Jiamei Lin, Liwen Feng, Tiantian Wang, Yuan Feng, Feng Yuan, Yang Mai, Xiaowei Zeng, Qi Zhang

**Affiliations:** 1 Cell-Gene Therapy Translational Medicine Research Center, The Third Affiliated Hospital of Sun Yat-Sen University, Sun Yat-Sen University, Guangzhou, 510630 China; 2 School of Pharmaceutical Sciences (Shenzhen), Shenzhen Campus of Sun Yat-Sen University, Sun Yat-Sen University, No. 66, Gongchang Road, Guangming District, Shenzhen, 518107 Guangdong China; 3 School of Biomedical Engineering, Shenzhen Campus of Sun Yat-Sen University, Sun Yat-Sen University, No. 66, Gongchang Road, Guangming District, Shenzhen, 518107 Guangdong China; 4 Boji Medical Biotechnological Co. Ltd, Boji Pharmaceutical Research Center, Boji Medical Building, No. 62 Nanxiang First Road, Science City, Huangpu District, Guangzhou, 510000 China; 5 Department of Hepatobiliary Surgery, The Third Affiliated Hospital of Sun Yat-Sen University, Sun Yat-Sen University, Guangzhou, 510630 China; 6 Department of Medical Oncology, The Third Affiliated Hospital of Sun Yat-Sen University, Sun Yat-Sen University, Guangzhou, 510630 China

## Correction to: Journal of Nanobiotechnology (2022) 20:90 https://doi.org/10.1186/s12951-022-01286-z

Following publication of the original article [1], the author has updated the funding information.

Guangzhou health care collaborative innovation major special project (201704020218).
