# Correction: Black phosphorus-Au-thiosugar nanosheets mediated photothermal induced anti-tumor effect enhancement by promoting infiltration of NK cells in hepatocellular carcinoma

**DOI:** 10.1186/s12951-025-03373-3

**Published:** 2025-04-15

**Authors:** Changchang Jia, Fan Zhang, Jiamei Lin, Liwen Feng, Tiantian Wang, Yuan Feng, Feng Yuan, Yang Mai, Xiaowei Zeng, Qi Zhang

**Affiliations:** 1 Cell-Gene Therapy Translational Medicine Research Center, The Third Affiliated Hospital of Sun Yat-Sen University, Sun Yat-Sen University, Guangzhou, 510630 China; 2 School of Pharmaceutical Sciences (Shenzhen), Shenzhen Campus of Sun Yat-Sen University, Sun Yat-Sen University, No. 66, Gongchang Road, Guangming District, Shenzhen, 518107 China; 3 School of Biomedical Engineering, Shenzhen Campus of Sun Yat-Sen University, Sun Yat-Sen University, No. 66, Gongchang Road, Guangming District, Shenzhen, 518107 China; 4 Boji Medical Biotechnological Co. Ltd., Boji Pharmaceutical Research Center, Boji Medical Building, No. 62 Nanxiang First Road, Science City, Huangpu District, Guangzhou 510000 China; 5 Department of Hepatobiliary Surgery, The Third Affiliated Hospital of Sun Yat-Sen University, Sun Yat-Sen University, Guangzhou, 510630 China; 6 Department of Medical Oncology, The Third Affiliated Hospital of Sun Yat-Sen University, Sun Yat-Sen University, Guangzhou, 510630 China

**Correction: Journal of Nanobiotechnology (2022) 20:90.**

10.1186/s12951-022-01286-z

In this article, Fig. 7 appeared incorrectly and has now been corrected in the original publication. For completeness and transparency, the incorrect and correct versions of Fig. 7 are displayed below.

Incorrect Fig. 7.

**Fig. 7:**
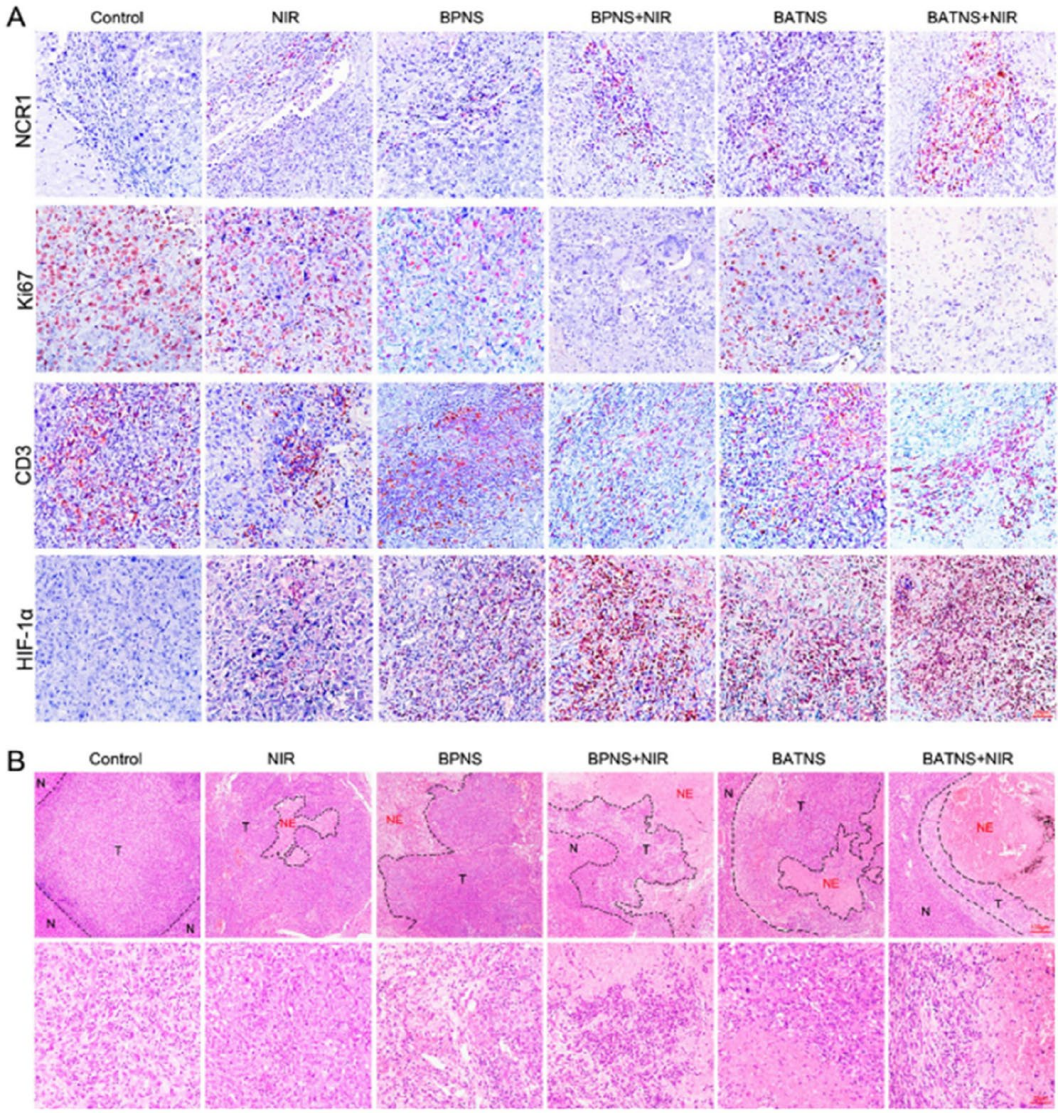
Analyze the changes of tumor histological indicators after BATNS photothermal treatment. **A** Immunohistochemical analysis of NK cells (NCR1), cell proliferation indicators (Ki67), T cells (CD3) and cellular hypoxia indicators (HIF-1α). **B** HE staining showed the local conditions of tumor tissue (T) adjacent to the cancer and necrotic tissue (NE) in the tumor in each experimental group. The tissue structure was observed under a ×100microscope (up), and the bottom under a ×400 microscope

Correct Fig. 7.

**Fig. 7:**
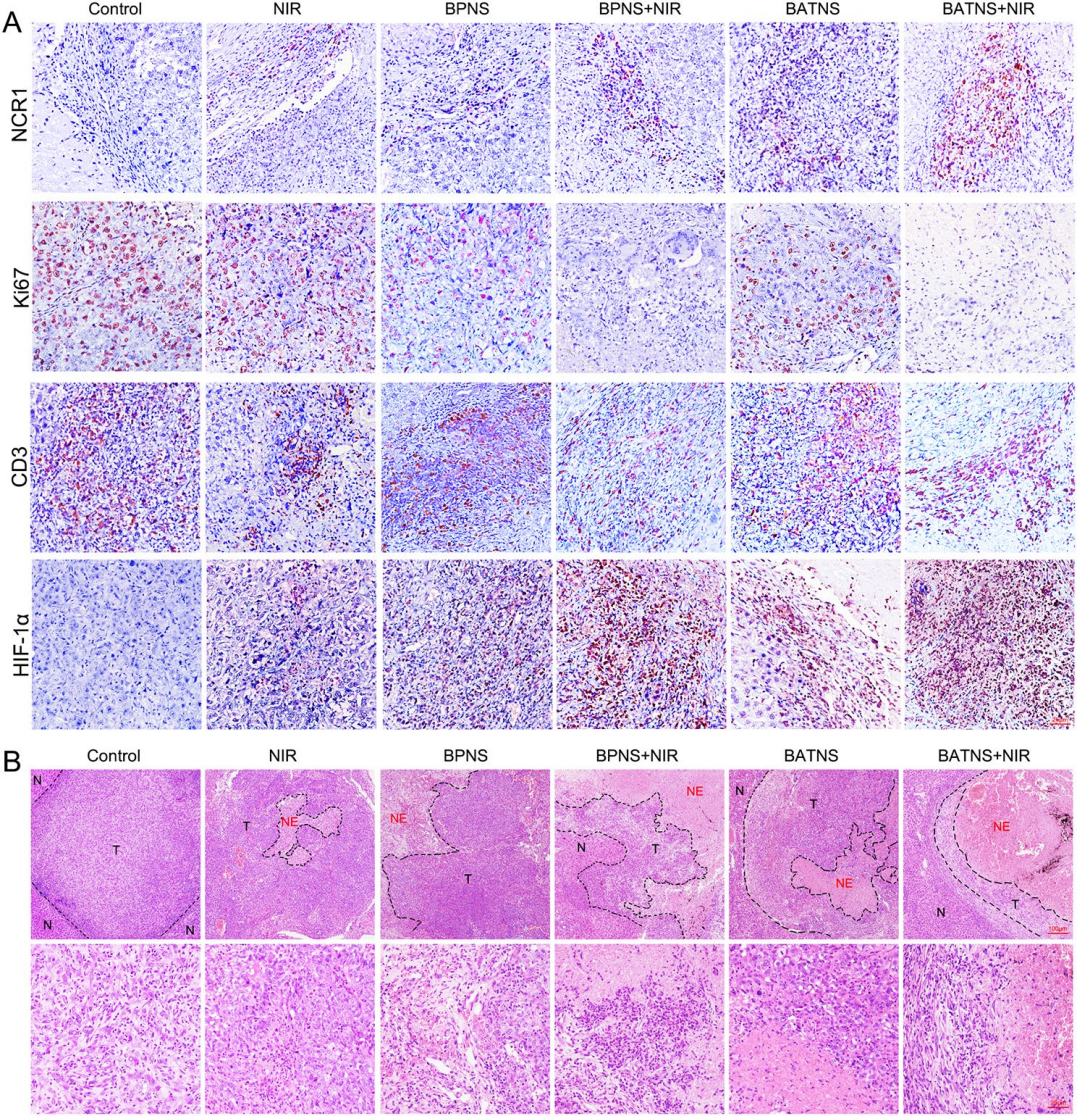
Analyze the changes of tumor histological indicators after BATNS photothermal treatment. **A** Immunohistochemical analysis of NK cells (NCR1), cell proliferation indicators (Ki67), T cells (CD3) and cellular hypoxia indicators (HIF-1α). **B** HE staining showed the local conditions of tumor tissue (T) adjacent to the cancer and necrotic tissue (NE) in the tumor in each experimental group. The tissue structure was observed under a ×100microscope (up), and the bottom under a ×400 microscope

The original article has been corrected.

