# Supplementary material for: Black phosphorus-Au-thiosugar nanosheets mediated photothermal induced anti-tumor effect enhancement by promoting infiltration of NK cells in hepatocellular carcinoma
Source: J Nanobiotechnology. 2022 Feb 21;20:90. doi: 10.1186/s12951-022-01286-z (PMC8862374; doi:10.1186/s12951-022-01286-z)
Supplement: Supplementary file 1 — Additional file 1: Figure S1. The chemical structure of auranofin. Figure S2. DLS size distribution of BPNS and BATNS. Figure S3. Zeta potential of BPNS and BATNS. Figure S4. AFM images of BPNS (A) and BATNS (B). AFM measured thickness of BPNS (C) and BATNS (D). Figure S5. EDS spectrum of BATNS. Figure S6. UV–vis absorption spectra of BPNS and BATNS in water and air for 120 h. Figure S7. Stability of BPNS and BATNS in water, respectively, by monitoring particle size for 7 days. Figure S8. Infrared thermal image of BATNS solution at different concentration (25, 50, 100, 200 μg/mL) with NIR laser irradiation (1.0 W/cm2, 10 min). Figure S9. Infrared thermal image of BATNS solution (50 μg/mL, 10 min) under the 808 nm NIR laser irradiation of various power (0.5, 1.0, 1.5, 2.0 W/cm2). Figure S10. Infrared thermal images of the tumor region injected with PBS, BPNS, BATNS under 808 nm NIR laser (1.0 W/cm2). Figure S11. (A) Flow cytometry analysis of the percentage of CD4+ and CD8+ cells in total CD3+ T cells. (B) Statistical analysis of the percentage of CD4+ and CD8+ cells in T cells in each experimental group. [file 12951_2022_1286_MOESM1_ESM.docx]

***Additional file 1***

**Black phosphorus-Au-thiosugar nanosheets mediated photothermal induced anti-tumor effect enhancement by promoting infiltration of NK cells in hepatocellular carcinoma**

Changchang Jia^1†^, Fan Zhang^3†^, Jiamei Lin^2†^, Liwen Feng^4^, Tiantian Wang^6^, Yuan Feng^5^, Feng Yuan^5^, Yang Mai^2*^, Xiaowei Zeng^2*^ and Qi Zhang^1*^

1.Cell-Gene Therapy Translational Medicine Research Center, The Third Affiliated Hospital of Sun Yat-sen University, Sun Yat-sen University, Guangzhou 510630, China

2.School of Pharmaceutical Sciences (Shenzhen), Shenzhen Campus of Sun Yat-sen University, Sun Yat-sen University, No. 66, Gongchang Road, Guangming District, Shenzhen, Guangdong 518107, China

3.School of Biomedical Engineering, Shenzhen Campus of Sun Yat-sen University, Sun Yat-sen University, No. 66, Gongchang Road, Guangming District, Shenzhen, Guangdong 518107, China

4. Boji Medical Biotechnological Co. Ltd., Boji Pharmaceutical Research Center, Boji Medical Building, No. 62 Nanxiang First Road, Science City, Huangpu District, Guangzhou 510000, China

5.Department of Hepatobiliary Surgery, The Third Affiliated Hospital of Sun Yat-sen University, Sun Yat-sen University, Guangzhou 510630, China

6.Department of Medical Oncology, The Third Affiliated Hospital of Sun Yat-sen University, Sun Yat-sen University, Guangzhou 510630, China

***** **Corresponding author:**

Qi Zhang, E-mail: zhangq27@mail.sysu.edu.cn

Xiaowei Zeng, E-mail: zengxw23@mail.sysu.edu.cn

Yang Mai, E-mail: maiy6@mail.sysu.edu.cn

**†These authors contributed equally to this work**

**

**

**Figure S1**. The chemical structure of auranofin.

**
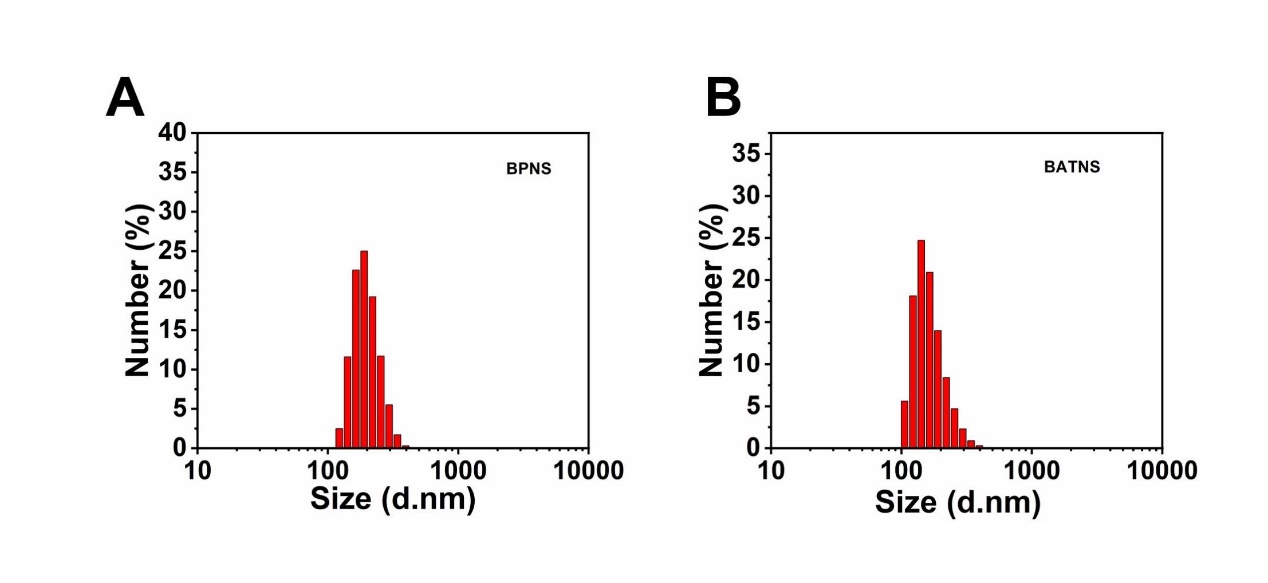
**

**Figure S2**. DLS size distribution of BPNS and BATNS.

**

**

**Figure S3**. Zeta potential of BPNS and BATNS.

**
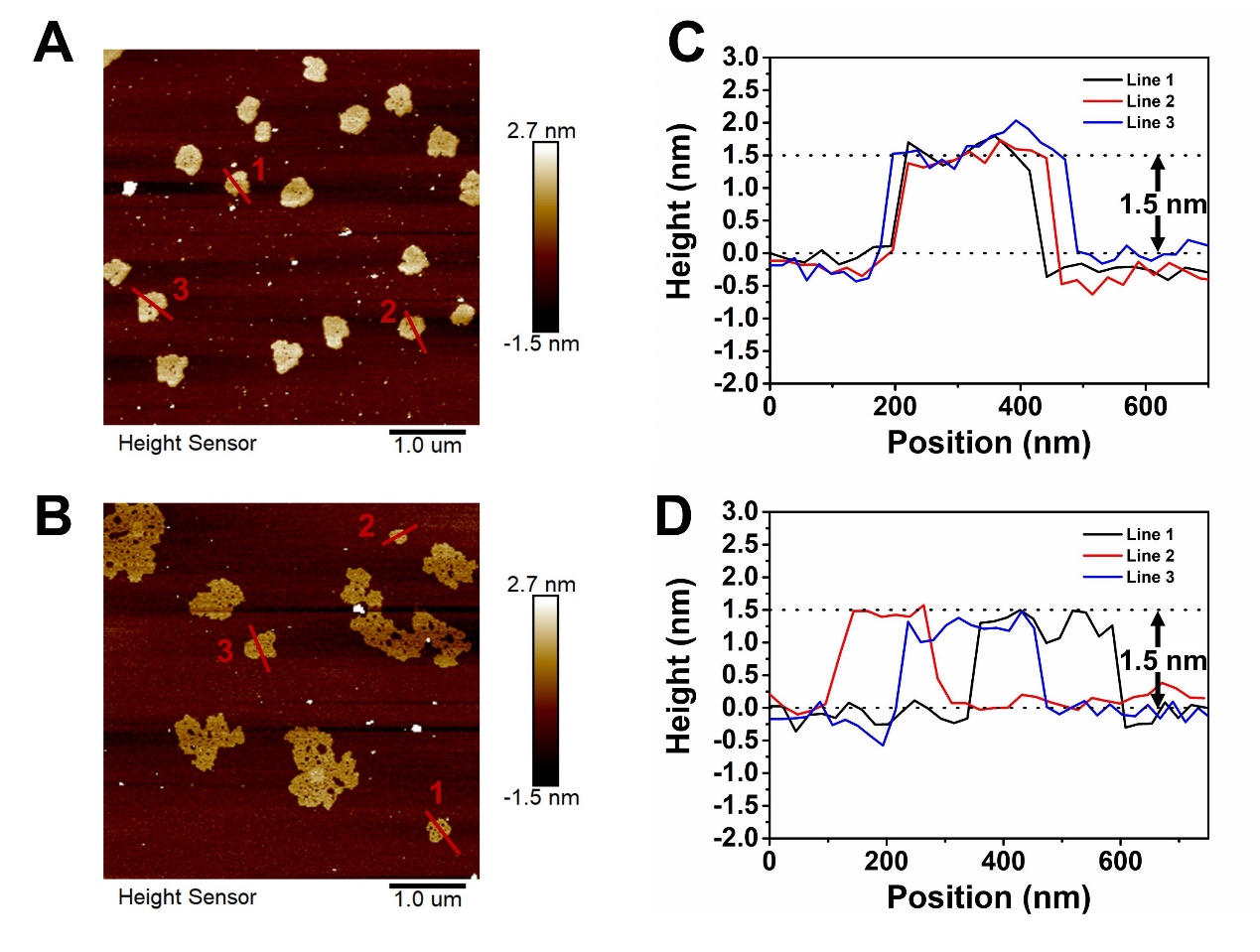
**

**Figure S4.** AFM images of BPNS (A) and BATNS (B). AFM measured thickness of BPNS (C) and BATNS (D).

**
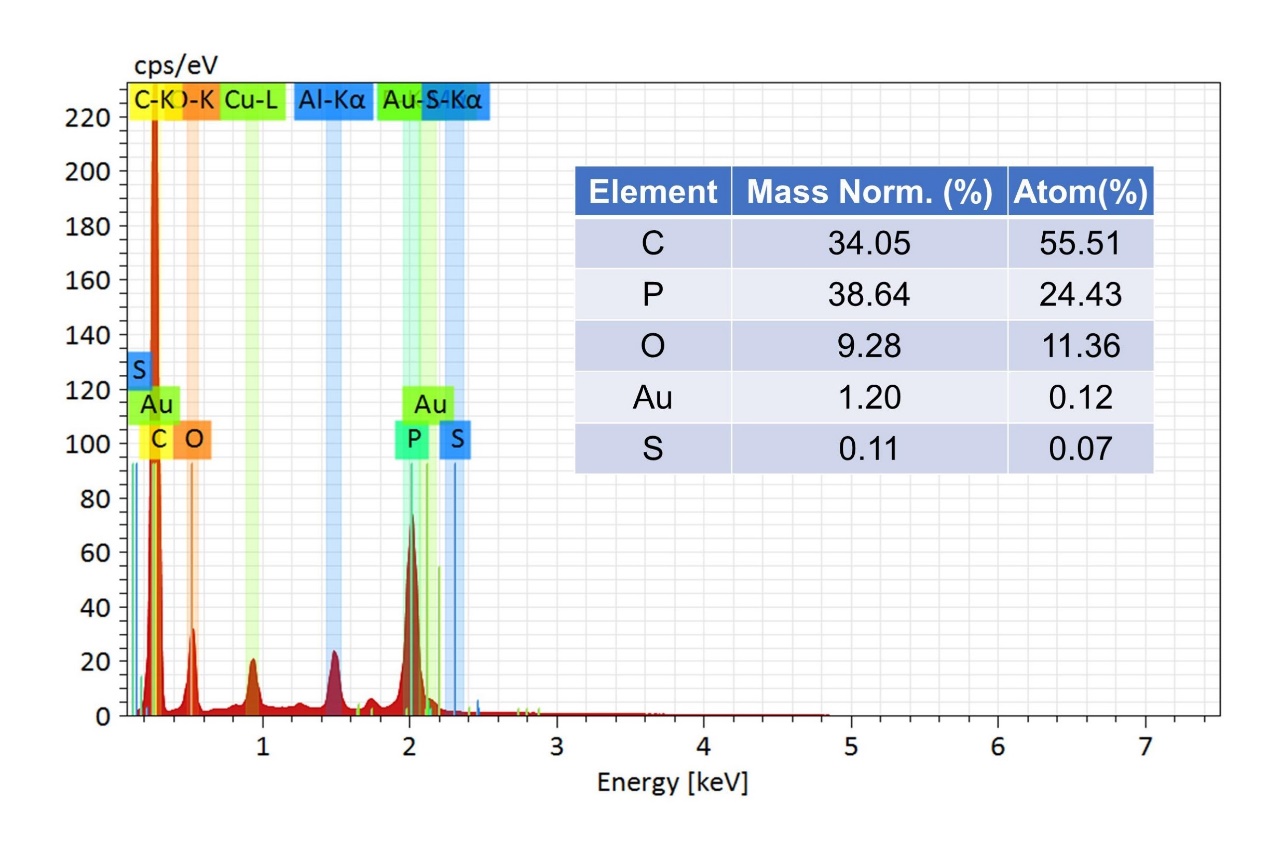
**

**Figure S5**. EDS spectrum of BATNS.

**
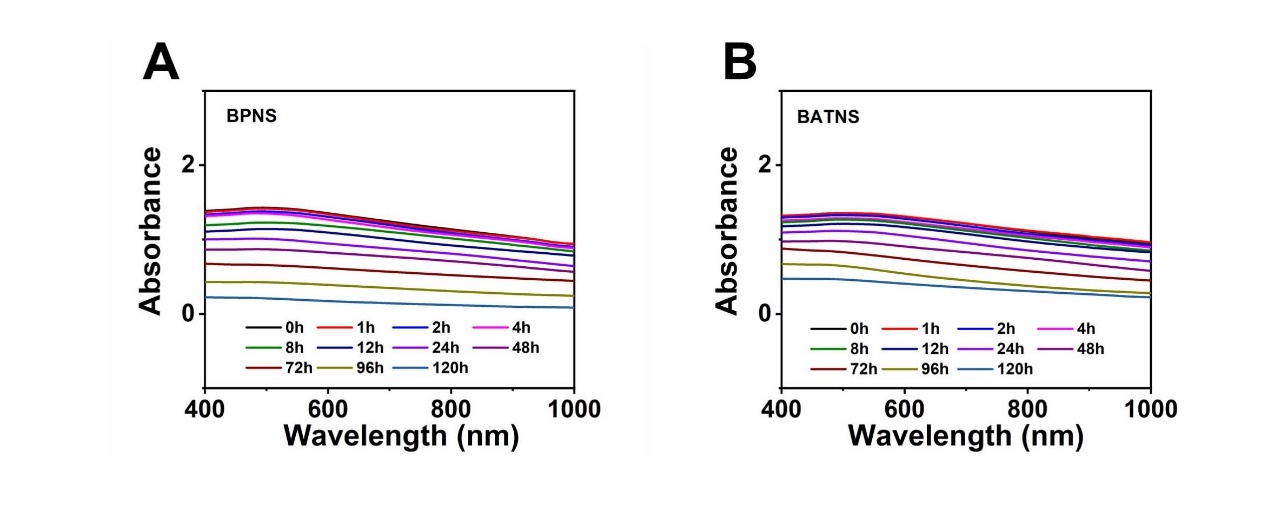
**

**Figure S6**. UV–vis absorption spectra of BPNS and BATNS in water and air for 120 h.

**
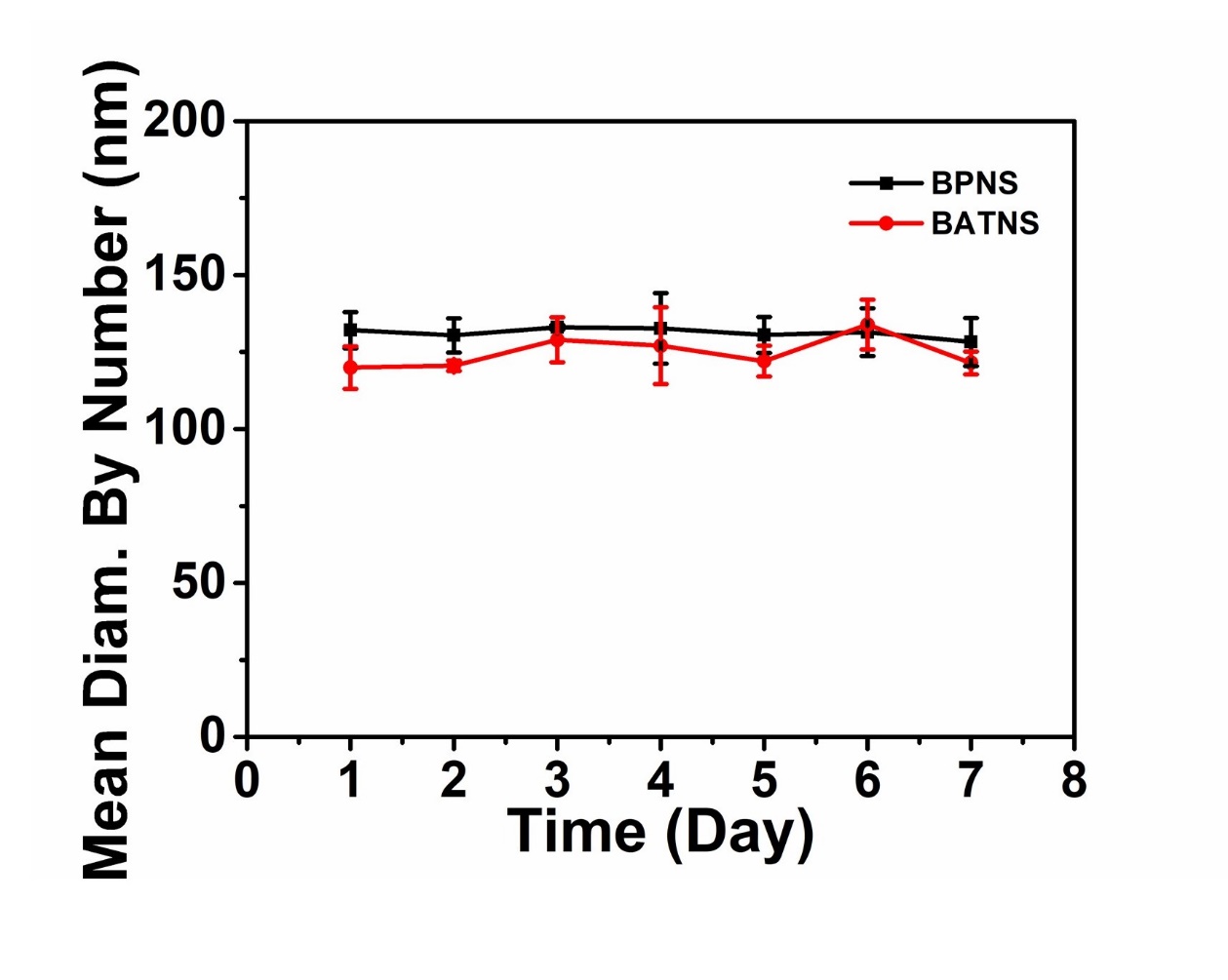
**

**Figure S7.** Stability of BPNS and BATNS in water, respectively, by monitoring particle size for 7 days.

**
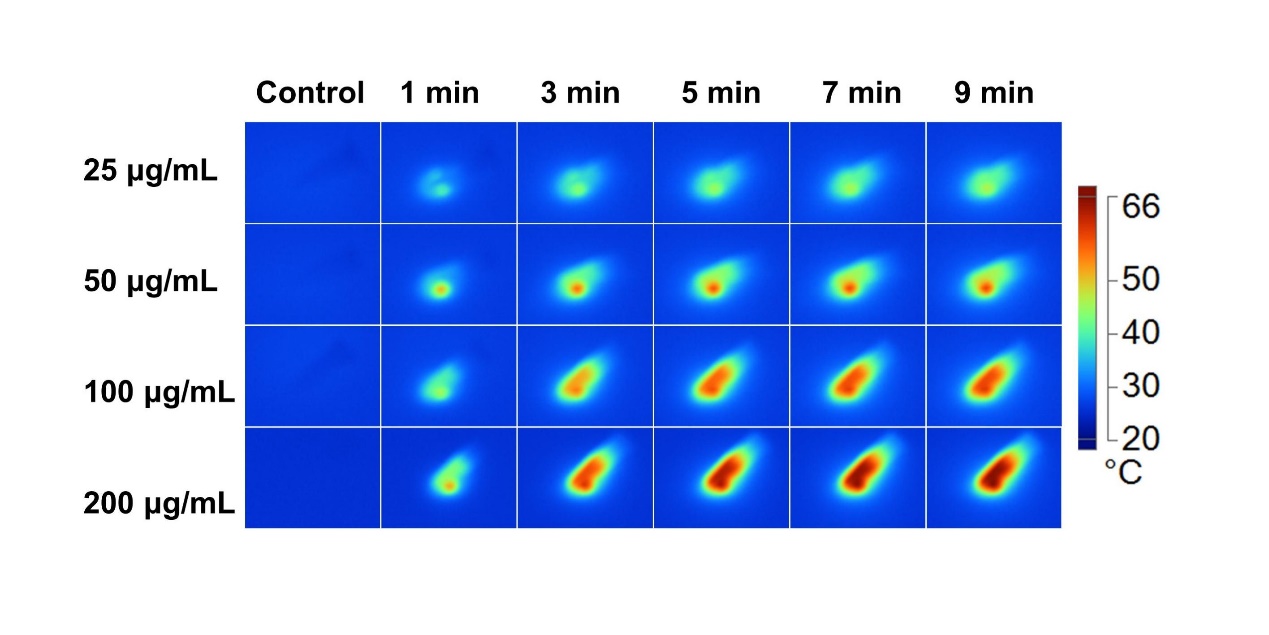
**

**Figure S8**. Infrared thermal image of BATNS solution at different concentration (25, 50, 100, 200 μg/mL) with NIR laser irradiation (1.0 W/cm^2^, 10 min).

**
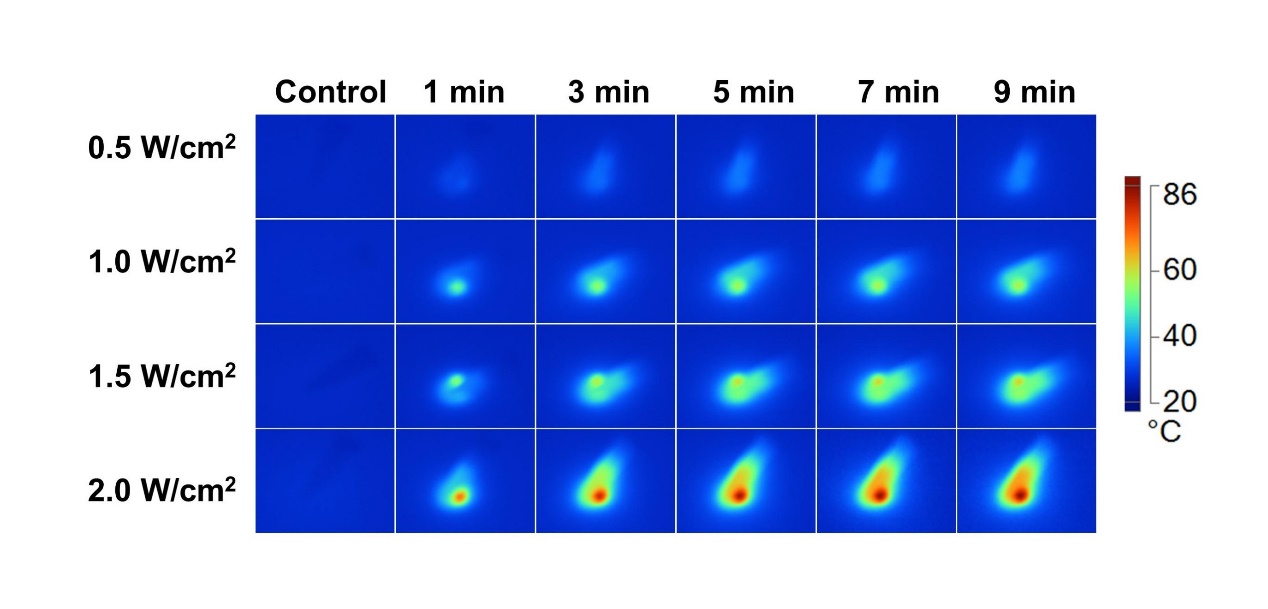
**

**Figure S9**. Infrared thermal image of BATNS solution (50 μg/mL, 10 min) under the 808 nm NIR laser irradiation of various power (0.5, 1.0, 1.5, 2.0 W/cm^2^).

**
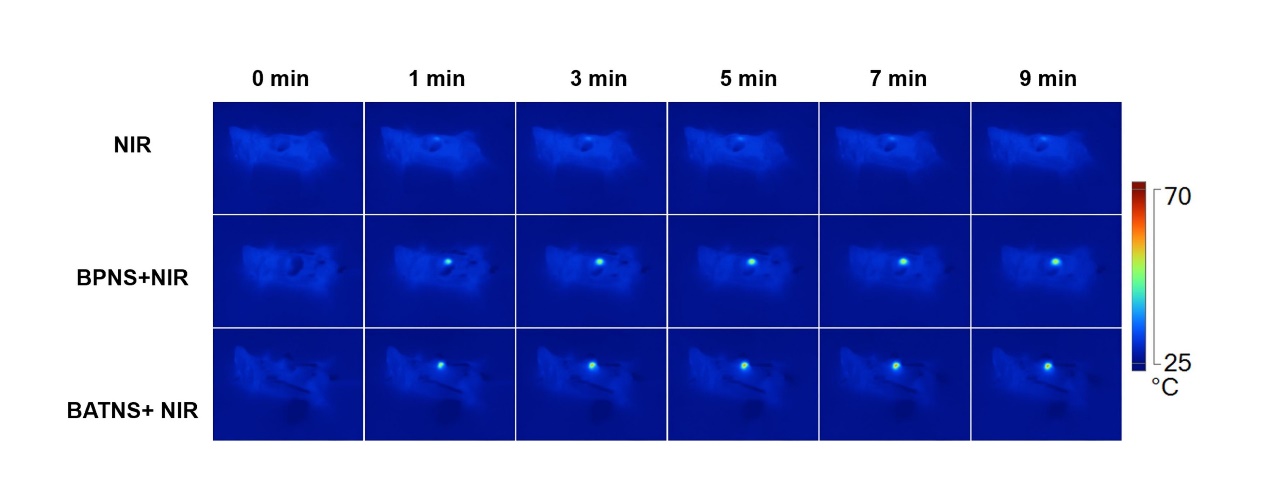
**

**Figure S10**. Infrared thermal images of the tumor region injected with PBS, BPNS, BATNS under 808 nm NIR laser (1.0 W/cm^2^).

**
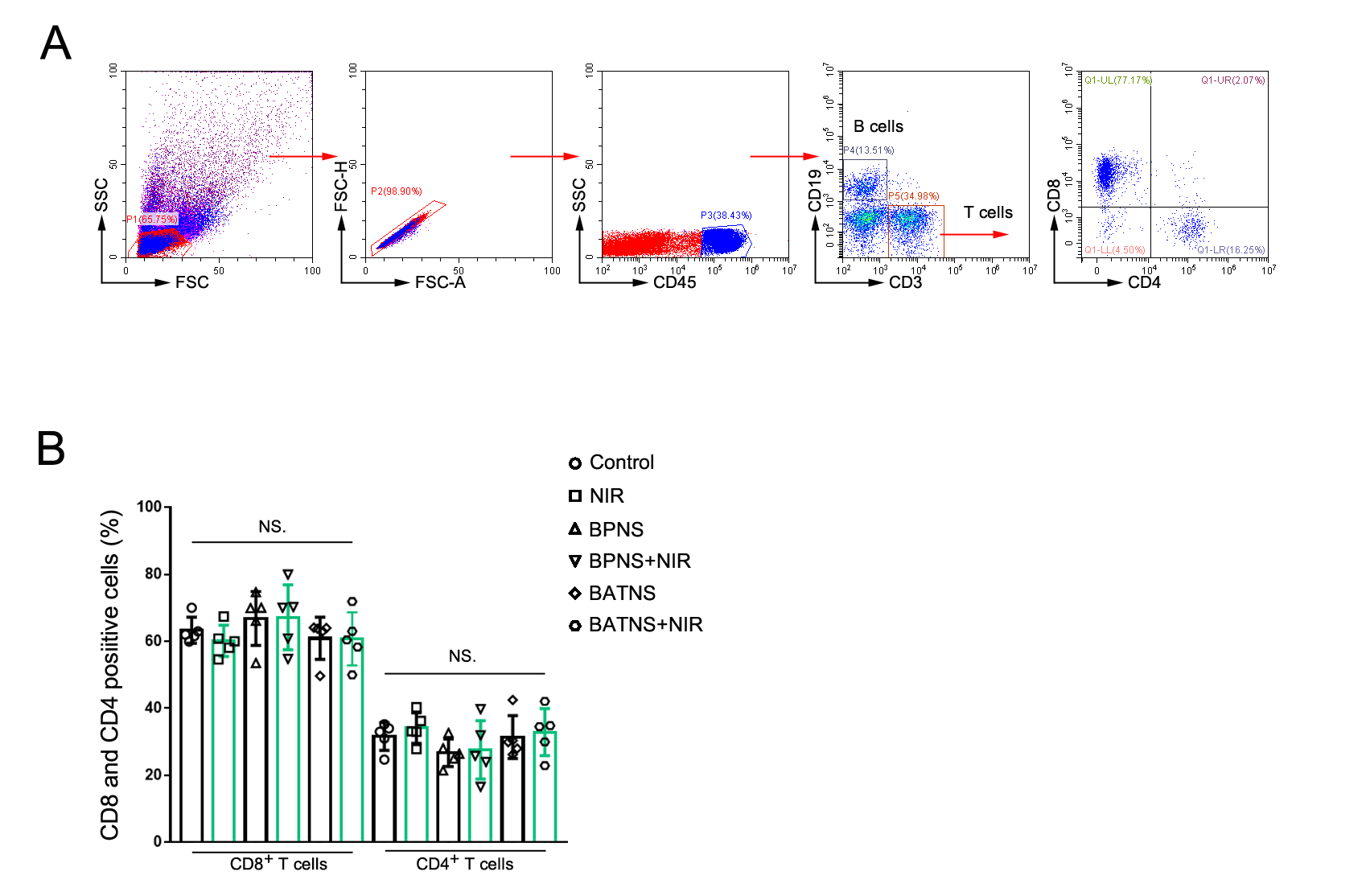
**

**Figure S11**. (A) Flow cytometry analysis of the percentage of CD4^+^ and CD8^+^ cells in total CD3^+^ T cells. (B) Statistical analysis of the percentage of CD4^+^ and CD8^+^ cells in T cells in each experimental group.
